# Triplet-Energy Quenching Functions of Antioxidant Molecules

**DOI:** 10.3390/antiox11020357

**Published:** 2022-02-11

**Authors:** Carlos Angelé-Martínez, Leticia Christina Pires Goncalves, Sanjay Premi, Felipe A. Augusto, Meg A. Palmatier, Saroj K. Amar, Douglas E. Brash

**Affiliations:** 1 Department of Therapeutic Radiology, Yale School of Medicine, New Haven, CT 06520-8040, USA; carlos.angele-martinez@yale.edu (C.A.-M.); leticiacpgoncalves@gmail.com (L.C.P.G.); sanjay.premi@moffitt.org (S.P.); felipe.augusto142@gmail.com (F.A.A.); meg.palmatier@yale.edu (M.A.P.); sarojkumaramar@gmail.com (S.K.A.); 2 Departamento de Química Fundamental, Instituto de Química, Universidade de São Paulo, São Paulo 05508-900, SP, Brazil; 3 Department of Dermatology, Yale School of Medicine, New Haven, CT 06520-8059, USA; 4 Yale Cancer Center, Yale School of Medicine, New Haven, CT 06520-8028, USA

**Keywords:** triplet-state, electronic excitation, chemiexcitation, chemiluminescence, dioxetane, energy transfer, cyclobutane pyrimidine dimer, antioxidant, triplet quencher, isomerization

## Abstract

UV-like DNA damage is created in the dark by chemiexcitation, in which UV-activated enzymes generate reactive oxygen and nitrogen species that create a dioxetane on melanin. Thermal cleavage creates an electronically excited triplet-state carbonyl whose high energy transfers to DNA. Screening natural compounds for the ability to quench this energy identified polyenes, polyphenols, mycosporine-like amino acids, and related compounds better known as antioxidants. To eliminate false positives such as ROS and RNS scavengers, we then used the generator of triplet-state acetone, tetramethyl-1,2-dioxetane (TMD), to excite the triplet-energy reporter 9,10-dibromoanthracene-2-sulfonate (DBAS). Quenching measured as reduction in DBAS luminescence revealed three clusters of 50% inhibitory concentration, ~50 μM, 200–500 μM, and >600 μM, with the former including sorbate, ferulic acid, and resveratrol. Representative triplet-state quenchers prevented chemiexcitation-induced “dark” cyclobutane pyrimidine dimers (dCPD) in DNA and in UVA-irradiated melanocytes. We conclude that (i) the delocalized pi electron cloud that stabilizes the electron-donating activity of many common antioxidants allows the same molecule to prevent an electronically excited species from transferring its triplet-state energy to targets such as DNA and (ii) the most effective class of triplet-state quenchers appear to operate by energy diversion instead of electron donation and dissipate that energy by isomerization.

## 1. Introduction

### 1.1. Triplet Energy and Triplet-Energy Quenching

The prevalence of melanoma and other skin cancers induced by ultraviolet radiation (UV) [1,2,3,4] has prompted the scientific community to search for new and more effective strategies for preventing photochemically induced diseases. Sunscreens and UV-filter clothing act by blocking UV from being absorbed by the DNA bases, thereby preventing an electron from being excited to the high energy that creates the mutagenic and carcinogenic cyclobutane pyrimidine dimer (CPD) [5,6,7].

Recently, we reported that CPDs are also generated long after UV exposure, by a physicochemical process termed “chemiexcitation” [8,9,10]. This mode of exciting an electron in DNA involves energy transfer from an excited reaction product of the oxidation of the skin pigment melanin. That prior excitation occurs in three steps. A brief UV exposure triggers sustained activity of the enzymes’ inducible nitric oxide synthase (iNOS) and NADPH oxidase (NOX), generating nitric oxide (NO^•^) and superoxide (O_2_^•−^) which react to create the strong oxidant peroxynitrite (ONOO^−^). Peroxynitrite oxidizes melanin, allowing O_2_ to add to the melanin and form a high energy 4-membered ring containing two adjacent O atoms, termed a dioxetane. The energy for this strained ring ultimately derives from the energy stored in O_2_ and the NO^•^ and O_2_^•−^ radicals [11,12]. Dioxetanes are unstable and thermally cleave to create two carbonyls, with one receiving nearly all the energy stored in the strained ring. Crucially, this energy is so high that one electron of a pair in the carbonyl double bond is excited to a new orbital; the energy also flips the spin of that electron, creating what is termed a “triplet state”. Because of the spin flip, decay to the ground state is “forbidden” by quantum mechanics and so takes seconds to minutes. In the interim, the long-lived triplet carbonyl can transfer its energy to a nearby acceptor molecule by exchanging its high energy electron for a ground state electron of the acceptor, a process termed “Dexter exchange” [13,14]. When the acceptor is DNA, the result can again be the cyclobutane pyrimidine dimer. The enzymatic activity generating O_2_^•−^ and NO^•^ persists for hours after activation by UV, continually generating new dioxetanes and forming new CPDs long after the UV exposure has ended, prompting the term “dark CPD” (dCPD) [8].

A new route to preventing dCPDs would then be to scavenge O_2_^•−^ and NO^•^. However, clinical trials of radical scavengers as cancer chemoprevention agents have encountered adverse effects [15,16], likely because radicals and ONOO^−^ are also elements of normal signaling pathways [17,18,19]. An alternative would be to dissipate the energy, using a triplet-energy quencher—a molecule that readily accepts triplet energy from a donor and then dissipates it as heat or light [20]. A ubiquitous example is the family of carotenoids, whose chain of conjugated double bonds provides delocalized electrons amenable to Dexter exchange; this strategy allows chloroplasts to dissipate excess triplet energy as heat [21].

In an initial survey for triplet quenching activity, we tested a variety of chemical structures derived from natural compounds and mixtures. The low risk/benefit profile of natural products makes them acceptable for use as preventive agents, where risk must be low for individuals who do not have a disease. In that survey (Table S1), we tested the ability of single or dual concentrations of compound to quench dCPDs or triplet-reporter luminescence resulting from chemiexcitation. It became apparent that: natural compounds often had substantial chemiexcitation-quenching activity; this activity was difficult to distinguish from ONOO^−^-scavenging activity; and for some natural compounds ONOO^−^ was able to generate dCPDs or reporter luminescence even using the compound itself as oxidizable substrate instead of melanin, although when combined with melanin the net effect was quenching of melanin chemiexcitation. We therefore focused on careful measurements of a small number of compounds using a synthetic triplet-state molecule not requiring ONOO^−^; we also broadened the range of structures similar to those that did have activity.

**Scheme 1:**
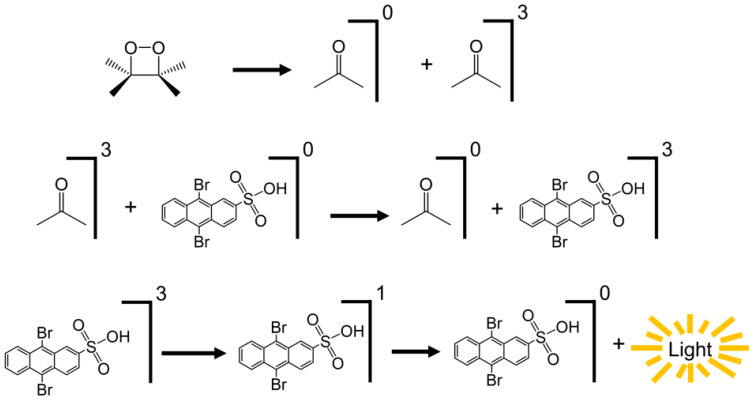
Sequence of reactions to produce luminescence as indicator of the formation of an excited triplet state. Triplet-state acetone derived from the decomposition of tetramethyl dioxetane (TMD) transfers energy to 9,10-dibromoanthracene-2-sulfonate (DBAS), which then emits light. 0, ground state; 1, singlet state; 3 triplet state.

To test biomolecules for triplet-quenching activity, we used triplet acetone (^3^A) as a model triplet-energy donor. The ^3^A was produced by the thermolysis of a synthetic dioxetane, tetramethyl dioxetane (TMD): TMD cleavage produces two molecules of acetone, with one being in the excited triplet state (Scheme 1) [22]. Triplet states are inefficient producers of luminescence, so to detect the triplet state we used the triplet-state reporter 9,10-dibromoanthracene-2-sulfonate (DBAS) [23]. Triplet acetone excites DBAS to a high-level triplet state which then converts to the excited S_1_ singlet state by intersystem crossing; the S_1_ state of DBAS is an efficient generator of fluorescence.

### 1.2. Polyphenols and Other Antioxidants

As candidate triplet-quenchers we sought natural compounds that, like carotenoids, have delocalized pi electrons. These arise from a conjugated double bond chain, an aromatic ring, or both. Candidate compounds also had absorbances in the UVB-UVA-visible range or shorter, indicating that they could accept energies from these levels or higher. Soluble compounds of this type were often already known for having antioxidant activity.

The radical scavenging activity of polyphenols results from their capacity to stabilize the radical formed after they donate an electron or H^•^ to free radicals. This stabilization occurs due to the multiple resonance structures across which the unpaired electron is distributed (delocalized) [24,25]. Another family of compounds, which protect cyanobacterial and aquatic plants (algae) from UV irradiation, are mycosporine-like amino acids (MAA) [26,27,28]. It has been proposed that, similarly to the polyphenols, MAA are synthesized from the shikimate pathway and protect against UV via their UV absorbance [29,30,31,32]. Some MAA exhibit radical scavenging activity and inhibit lipid peroxidation [33,34,35]. In addition, some MAA are triplet-state energy acceptors [36,37,38,39].

**Figure 1:**
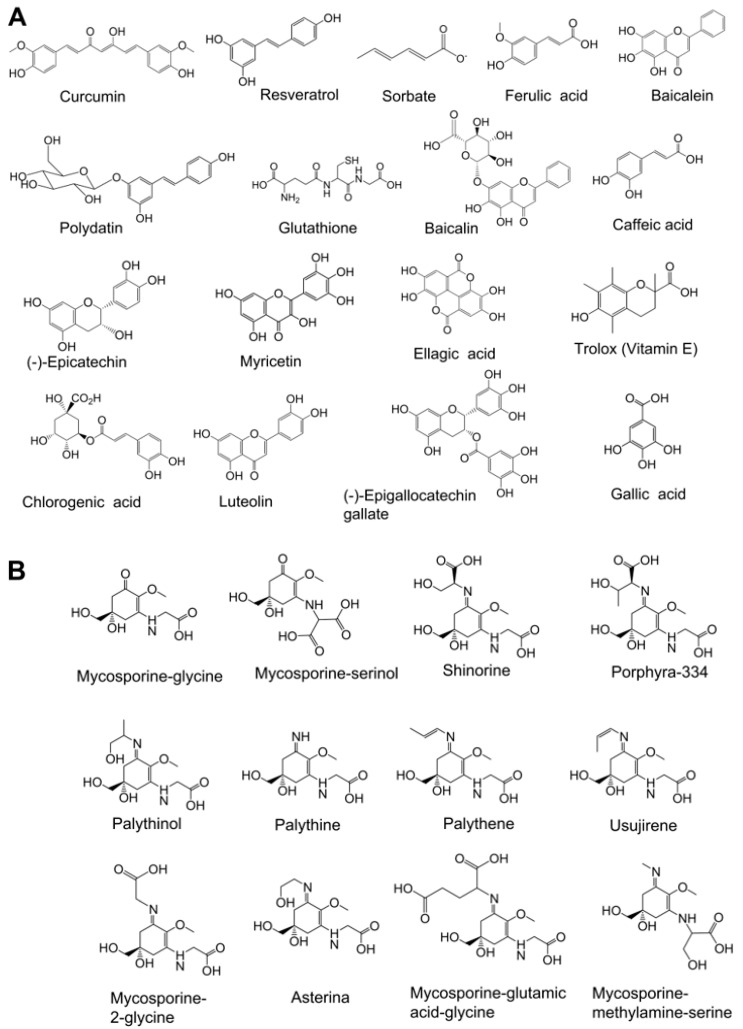
Molecular structure of compounds used to evaluate triplet-state quenching. (**A**) Polyphenols and related compounds featuring delocalized electrons; (**B**) Mycosporine-like-amino acids present in algae extracts.

We therefore selected more than 15 polyphenols, related compounds, and mixtures of these (Figure 1A), as well as 45 algae extracts from species known to synthesize MAA, Figure 1B). From the results of this analysis, we chose representative compounds for analyzing the capacity to prevent dCPD formation.

## 2. Materials and Methods

### 2.1. Assay Design and Specificity

In a cell, using dCPDs as a screening endpoint would be blocked not only by true triplet-state quenchers but also by false positives that do one of the following:

- Absorb UV.
- Block UV-activation of the NADPH oxidase or nitric oxide synthase that make O_2_^•−^ and NO^•^.
- Scavenge O_2_^•−^, NO^•^, or ONOO^−^.
- Prevent the dioxetane substrate from reaching the nucleus (by blocking melanin fragmentation or migration).
- Shield the dioxetane substrate (by altering the melanin reactive site, perhaps by altering fragmentation by generating ^1^O_2_).
- Block dioxetane creation at a post-ONOO^−^ step.
- Are decoy substrates for a dioxetane (yet are poor at making dCPDs).
- Cleave CPDs by electron donation or withdrawal [40,41,42].

These false positives are avoided by using TMD and the DBAS assay. Molecules that prevent or absorb acetone phosphorescence or DBAS fluorescence would be a false positive in the DBAS assay, but are avoided in the CPD assay because their 440 nm luminescence [23,43] will not make CPDs.

Using both assays, hits are molecules that do one of the following:

- Cleave a dioxetane unproductively, by electron donation [44,45,46].
- React covalently with the triplet carbonyl [47].
- Accelerate the triplet state’s intersystem crossing to the singlet, via partial charge transfer [48].
- Block Dexter electron exchange, by electron donation that occupies the ground-state exchange site (see Section 4.6.2).

Evidence for energy transfer would be evidence against those four mechanisms. One possibility remains, that hits are molecules that:

- Act as decoy triplet-energy transfer targets (absorbing triplet energy better than DBAS or DNA while not generating luminescence or dCPDs, although luminescence would be a safe mode of energy dissipation). These are physical triplet-state quenchers.

Complete methods for the screens are given in Supplemental Methods.

### 2.2. Quenching of Triplet-Reporter Luminescence by Polyphenols and Related Compounds

TMD-DBAS-polyphenol chemiluminescence assays were performed in 300 μL total reaction volume. Samples were prepared by mixing the phosphate buffer (pH 7, 25 mM), DTPA (0.2 mM), the natural compounds solution at various concentrations (0.05–15,000 μM), TMD (1 mM), and the DBAS (1 mM). DTPA was used as the metal chelator because metal ions catalyze the decomposition of TMD [45]. Samples were aliquoted (100 μL) in duplicate into white 96-well plates Greiner Lumitrac 200 (Fisher Scientific, Pittsburgh, PA, USA) and the chemiluminescence was measured in a FluoDia T70 Microplate Photon Counting Fluorometer (Photal Otsuka Electronics, Osaka, Japan), using 300 ms as the integration time, and 73 °C as the nominal temperature. 

### 2.3. Quenching of Dark Cyclobutane Pyrimidine Dimer Formation

In a microcentrifuge tube, 1 μg of dT_150_ or calf thymus DNA (^CT^DNA) in phosphate buffer (pH 7, 50 mM) and DTPA (0.5 mM) was treated with TMD (5 mM) and freshly prepared solutions of epicatechin, potassium sorbate, or luteolin (10 μM) in a total volume of 200 μL. The solution with all reagents was vortexed briefly and incubated at 70 °C for 10 min. The samples were purified by column elution. To assess triplet-quenching in cells, C57BL/6 murine melanocytes were exposed to 75 kJ/m^2^ UVA as previously reported [8], 100 μM quencher added, and the cells harvested at 2 h. 

### 2.4. Detection of Cyclobutane Pyrimidine Dimers by Enzyme Linked Immunosorbent Assay (ELISA)

#### 2.4.1. Denaturation and Plating of dT_150_ and ^CT^DNA

From samples treated with TMD and column purified, or UVC irradiated, a 3 ng/μL solution (160 μL) was prepared. The samples were denatured at 95 °C for 15 min and then transferred to an ice-water bath for 15 min. The samples were aliquoted in triplicate (50 μL) into wells of a 96-well non-binding Greiner plate (E19033A5) that had been pre-washed six times with TBS (1X), Tween (0.05%, see above) and then three times with deionized H_2_O. The plate was incubated at 37 °C overnight.

#### 2.4.2. Antibody Incubation and Visualization

The plate was washed five times with Tris buffered saline-Tween solution (TBST 1X), discarding the buffer and wiping the plate onto an absorbing pad after each wash. The plate and the oligo were blocked with 300 μL of 2% NGS in Tris buffered 1X TBS by incubating the solution at 37 °C for 30 min. The NGS solution was discarded and the plate wiped. 

Primary antibody, TDM-2, in TBS (1X) was added (100 μL) to each plated sample well and incubated at 37 °C for 30 min. The plate was washed five times with TBST (1X), discarding the buffer and wiping the plate onto an absorbing pad after each wash. Secondary antibody in TBS (1X) was added (100 μL) to the plated samples and incubated at 37 °C for 30 min. The plate was washed five times with TBST (1X) and once more with carbonate buffer, with the buffer discarded, and the plate wiped onto an absorbing pad after each wash.

One hundred microliters of Dynalight solution (Thermo Fisher Scientific, Dynalight Substrate with RapidGlow Enhancer, 4475406) were added to the plated samples and incubated at room temperature for 30 min. Fluorescence of the samples was measured in a FluoDia T70 Microplate Photon Counting Fluorometer (Photal Otsuka Electronics), using 300 ms as integration time at room temperature.

### 2.5. Calibration Curve for Quantifying CPD Generated by Chemiexcitation

The CPD ELISA gives a nonlinear dose response, so ELISA values were converted to physical values related to CPD density. We used UVC to create DNA samples with a known and well-characterized series of CPD densities and having a minimum of other DNA lesions. A solution of dT_150_ (10 ng/μL, 2.5 mL) was prepared from the solid stock. The solution was split into 10 μL drops on Parafilm on a cold block. The drops were irradiated with a UVC lamp calibrated with a UVX Radiometer (Analytik Jena, Jena, Germany, UVP 97001502) and a UVC probe (Analytik Jena, UVX-25) for a time providing a dose of 100 J/m^2^. After irradiation, the sample was column purified as in Section 2.5. Similarly, a non-irradiated solution of dT_150_ (10 ng/μL, 8 mL) was column purified. The concentration of purified solutions was measured in duplicate (1.5 μL) by Nanodrop as above. Both solutions were diluted to 3 ng/μL and the irradiated solution was mixed with the non-irradiated solution at different ratios to provide dT_150_ equivalent to irradiation at different irradiation doses. These calibration standards were used within each experiment to generate ELISA calibration curves. The curves were used to convert ELISA values to their UVC Equivalent dose (in J/m^2^). A UVC dose of 5 J/m^2^, the approximate value seen in the 0 hr controls, generates ~1 CPD per 10 kbp [49].

### 2.6. Quantification of Quenching

#### 2.6.1. Biochemical Studies

To calculate IC_50_ values for inhibiting TMD-DBAS luminescence, the average luminescence signal of the non-chemiluminescent wells containing no TMD or DBAS was subtracted from the chemiluminescence signal of each experimental well containing TMD, DBAS, and the putative quencher, with the result denoted S_TDQ_. The luminescence from TMD or DBAS alone was not significant. The Percent Chemiluminescence was defined as the ratio of luminescence in the presence of quencher to that in the absence of quencher, 100 * (S_TDQ_/S_TD_). Luminescence inhibition is then 100—S_TDQ_/S_TD_. The IC_50_ value was calculated by plotting % Chemiluminescence versus the logarithm of the concentration of the triplet quencher concentration (in μM) and fitting to a variable slope sigmoidal dose-response curve using SigmaPlot version 11.0 (Systat Software, Inc., San Jose, CA, USA). Average IC_50_ values and IC_50_ experimental errors reported in Table 1 are calculated from at least three independent experiments. The percent CPD inhibition was calculated as 100% CPD increase. Because the DNA-alone ELISA signal was not negligible, % CPD increase was calculated as 100 * (S_TDQ_ − S_D_)/(S_TD_ − S_D_) and D now stands for DNA alone rather than DBAS. The standard deviation of the quenching was calculated from two independent experiments. 

#### 2.6.2. Cell-Based Studies

The scale of CPD production at 0 h and of dCPD production at 2 h both varied between experiments. Cell-based UVC Equivalent dose was therefore normalized to the value at 0 h. The pre-normalized data are presented in Figure S20.

## 3. Results

### 3.1. Quantification of Triplet-State Quenching Using Triplet-State Reporter Luminescence

**Figure 2:**
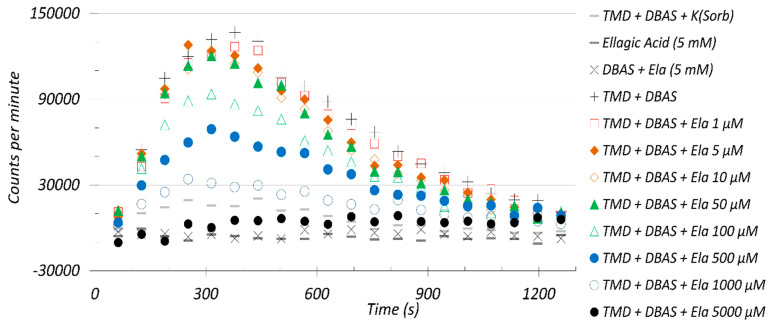
Quenching of tetramethyl dioxetane induced luminescence of 9,10-dibromoanthracene-2-sulfonate (DBAS) by various concentrations of ellagic acid (Ela, 0–5000 μM).

**Figure 3:**
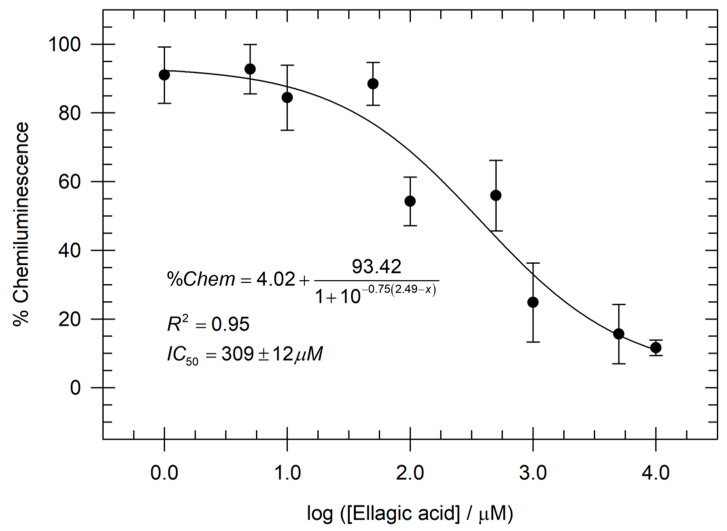
IC_50_ curve of ellagic acid calculated from the percentage TMD-DBAS luminescence reduction versus logarithm of ellagic acid concentration.

Triplet-quenching capacity was measured for seventeen polyenes, polyphenols, and related compounds, as well a mixture of these. Potassium sorbate (a known triplet-state quencher) [23] was used as control, to demonstrate the adequacy of the experimental conditions and as a reference standard. The triplet-state carbonyl was formed by heating the samples containing TMD, DBAS, and the triplet quencher to 73 °C in the same instrument in which the chemiluminescence was measured. Figure 2 shows the TMD-DBAS chemiluminescence in the presence of ellagic acid at different concentrations and at different times after initiating the reaction. The chemiluminescence signal decreased as the concentration of ellagic acid increased. Based on this result, the concentration of ellagic acid required to quench 50% of the TMD-DBAS chemiluminescence (IC_50_) was calculated: the signal from each ellagic concentration at 400 s (maximum signal) was divided by the TMD-DBAS-only signal; this ratio was plotted (as a percentage) with respect to the logarithm of test compound concentration (Figure 3). The curve provides a quantifiable measure of how effective the polyphenol is at preventing triplet-energy state generation from the TMD-DBAS excitation. The IC_50_ value was calculated for each of the polyphenols and other compounds of interest (Figures S1–S17). 

### 3.2. Three Categories of Triplet-State Quenching Activity

We found that the compounds fell into three groups based on the IC_50_ values (Table 1). The first group were the compounds with excellent triplet-quenching activity (29–150 μM): curcumin (29 μM), resveratrol (52 μM), potassium sorbate (58 μM), and ferulic acid (129 μM). The second group had moderate triplet quencher activity: baicalein (195 μM), polydatin (resveratrol with a sugar, 234 μM), glutathione (234 μM), baicalin (baicalein with a sugar, 251 μM), caffeic acid (251 μM), epicatechin (288 μM), myricetin (309 μM), and ellagic acid (309 μM). The third group had triplet quencher activity but only at higher concentrations: trolox (427 μM), chlorogenic acid (caffeic acid with a sugar, 550 μM), luteolin (~600 μM), epigallocatechin gallate (698 μM), and gallic acid, 708 μM). 

**Table 1:** Concentration range and IC_50_ value of natural compounds that quench triplet-state energy, assessed by the luminescence of TMD-DBAS energy transfer.

Group	Polyphenol	Luminescence Quenching Range (μM)	IC_50_ Value (μM)
I	Curcumin	0.05–15,000	29 ± 1
	Resveratrol	0.05–15,000	52 ± 2
	Potassium Sorbate	0.1–5000	58 ± 5
	Ferulic Acid	1–15,000	129 ± 11
II	Baicalein	0.05–15,000	195 ± 14
	Polydatin	1–15,000	234 ± 18
	Glutathione	1–10,000	234 ± 11
	Baicalin	0.1–5000	251 ± 20
	Caffeic Acid	0.05–15,000	251 ± 16
	Epicatechin	0.1–10,000	288 ± 21
	Myricetin	1–5000	300 ± 23
	Ellagic Acid	1–10,000	309 ± 12
III	Trolox (Vitamin E)	0.05–15,000	427 ± 20
	Chlorogenic acid	0.05–15,000	550 ± 15
	Luteolin *^a^*	500–5000	~600
	Epigallocatechin Gallate	0.1–10,000	698 ± 21
	Gallic Acid	0.05–15,000	708 ± 47

*^a^* Luteolin enhances the chemiluminescence of the samples in the concentration range of 1–50 μM.

Table 2 shows the IC_50_ values of a natural product mixture, given in μg/mL. It is imprecise to calculate the molar concentration of mixtures because they are multicomponent and the percentage of each compound is not known. Thus, for comparison, we included the IC_50_ value in these units for potassium sorbate, chlorogenic acid, and epigallocatechin gallate. The similarity of the components of Honeysuckle extract (IC_50_ = 245 μg/mL, Figure S18) to epigallocatechin gallate (Table 2 and Figure 1) is reflected in their IC_50_ values (IC_50_ = 320 μg/mL for epigallocatechin gallate). The presence of multiple compounds in a mixture makes it difficult to relate the activity directly to only one compound. The activity of honeysuckle extract was ~25% lower than chlorogenic acid (that is, had a larger IC_50_), suggesting that the presence of more than one component in the test sample results in a higher IC_50_ value than with single compounds. The same may have occurred with luteolin and epigallocatechin gallate, which were only ≥95% pure. Honeysuckle extract also has the particularity that its polyphenolic compounds (Table 2) are bound to a sugar moiety (Figure 1), which evidently depresses the triplet quenching activity as observed in Table 1 for polydatin, baicalin, and chlorogenic acid compared to resveratrol, baicalein, and caffeic acid, respectively.

### 3.3. Algal Extracts

Forty-five algae extracts were also analyzed to evaluate their capacity to quench triplet acetone. Species were chosen based on their reported propensity to contain mycosporine-like amino acids, commonly studied for their UV-absorbing properties [26,28,30,31,50,51]. Due to the large number, only two concentrations of each algae extract were tested. Table 3 and Figure S18 show the results for the six most effective extracts (see Supplemental Table S2 for results of all 45 extracts). Despite the impossibility of calculating an IC_50_, it is clear that the extracts contain triplet quenching activity. 

### 3.4. Quenching of CPD Formation

After observing that most of the tested compounds quenched TMD-generated triplet-state acetone, as judged by preventing luminescence from the triplet-state reporter DBAS, we chose one triplet quencher from each group of Table 1 to evaluate their capacity to prevent the formation of dark CPDs. First, we found that TMD does induce dCPDs in DNA, as reported for trimethyl dioxetane [52]. Samples of a 150-mer of thymine (dT_150_) or calf thymus DNA (^CT^DNA) were mixed with 5 mM TMD and incubated at 70°C to induce dioxetane cleavage and triplet-state carbonyls. The dCPDs were measured by ELISA and a standard curve was used to express the CPD level as an equivalent UVC dose, which, unlike ELISA, is a linear measurement (see Methods). TMD treatment increased the CPD signal of dT_150_ and ^CT^DNA 1.3- and 1.6-fold, respectively, relative to the signal without TMD. This increase is in the range previously seen in cells, where in the absence of nucleotide excision repair the dCPDs constitute approximately half the CPDs received after UV exposure [8]. The absolute magnitude was equivalent to ~1 J/m^2^ UVC; this dose is slightly toxic in mammalian cells after a single exposure, but a continuing chemical source of such levels would produce a cumulative effect. When potassium sorbate, epicatechin, or luteolin were added to the dT_150_ or ^CT^DNA samples treated with TMD, dCPDs were inhibited up to 90% even at quencher concentrations (10 μM) well below their IC_50_ in the DBAS reporter assay (Table 4). The trend in effectiveness of the compounds in calf thymus DNA reflects their IC_50_; however, dT_150_ had the opposite behavior, perhaps reflecting a property of single-strand DNA or thymine. 

### 3.5. CPD Quenching in UVA-Irradiated Melanocytes

**Figure 4:**
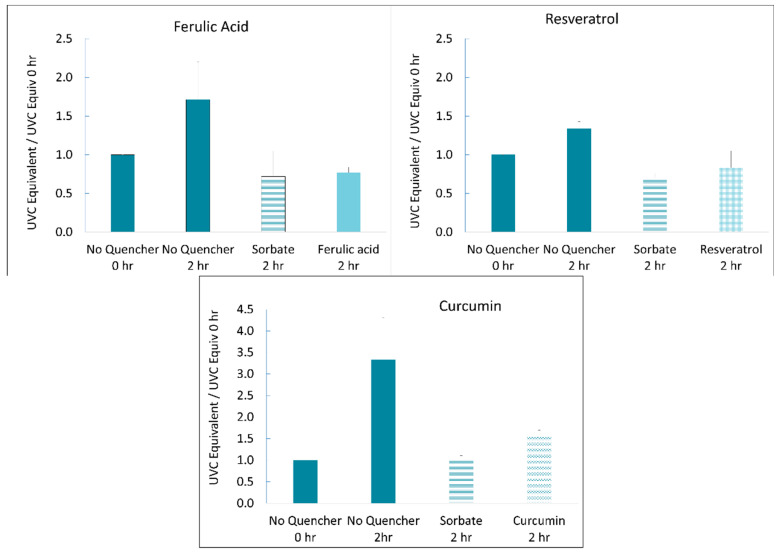
Quenching of chemiexcitation-induced dark cyclobutane pyrimidine dimers (dCPD) in C57BL/6 melanocytes by triplet-state quenchers. Chemiexcitation was induced by 75 kJ/m^2^ UVA, which directly induces the level of CPDs observed at 0 h. At the end of UVA exposure, 0 or 100 μM test compound was added. “Dark CPDs” are generated during the 2 h after exposure (and some may be generated during the period of UVA exposure). The presence of a quencher reduced the CPD level to that of 0 h direct CPDs. Due to the non-linearity of the ELISA assay, ELISA OD_490_ signals are calibrated against UVC-irradiated DNA to give UVC Equivalent doses that are proportional to CPD per kb (see Methods and Figure S20). Values are normalized to the 0 h UVC Equivalent dose to compensate for baseline variations between experiments. A ratio of 1.0 typically corresponded to a UVC dose of 5 J/m^2^ to purified DNA, which generates ~1 CPD per 20 kbp and is lethal to ~30% of a population of human cells [49,53]. A value of 1.5, corresponding to ~7.5 J/m^2^ UVC, is ~70% lethal. Combining results from all experiments gives *p* = 0.03 for the difference between 0 h and 2 h without quencher; *p* = 0.02 for the difference between ±sorbate; and *p* = 0.01 for the effect of the other quenchers pooled.

Four compounds highly effective in quenching TMD-DBAS luminescence were tested for their ability to prevent dCPD formation in murine melanocytes when added after UVA exposure, a wavelength that makes fewer direct CPDs than UVC or UVB but was effective in inducing dCPDs [8]. Potassium sorbate, a more soluble analog of the ethyl sorbate used in the previous study, was used as positive control. Despite cell uptake being an additional factor in the in vivo assay, sorbate, ferulic acid, resveratrol, and curcumin were each effective at 100 μM in limiting dCPD formation observed 2 h after 75 kJ/m^2^ UVA (Figure 4). The CPD levels with quencher resembled the level of directly induced CPDs at 0 h. The cell-based results alone would not distinguish between quenching dCPDs and accelerating repair of all CPDs, although the typical half-time for CPD repair in human cells is 24 h. The DNA-based data in Table 4 are consistent only with quenching. 

## 4. Discussion

### 4.1. Triplet-State Quenching by Antioxidant Biomolecules

Structural properties of these quenchers suggest distinct mechanisms of quenching. Exclusion of false positive mechanisms not of interest was described in Section 2.1 of Methods. For the true positives, we first consider physical quenching—in which the number of electrons, bonds, or atoms of the triplet quencher does not change—and we then consider chemical modes of triplet quenching in Section 4.6. 

Physical energy quenching entails two steps: energy transfer then energy dissipation. The Introduction mentioned that the delocalized electrons in these compounds support energy transfer by Dexter electron exchange and that the same property supports radical scavenging activity. We now point out several known mechanisms of energy dissipation, based on published experiments using UV light as the source of the excitation energy.

### 4.2. Triplet-Energy Dissipation by Trans-cis Isomerization

All the compounds of Group I (curcumin, resveratrol, sorbate, ferulic acid) have at least one *trans* double bond that can undergo isomerization to *cis*. In this structure, absorption of a photon or transfer of energy from a triplet state is known to excite an electron of the triplet quencher, twisting the double bond and converting it to a diradical having a single bond around which the molecule can now isomerize [54,55,56]. Isomerization dissipates the triplet energy as heat due to collisions with the solvent or, if the final isomer has higher energy, as ground-state bond energy.

**Figure 5:**
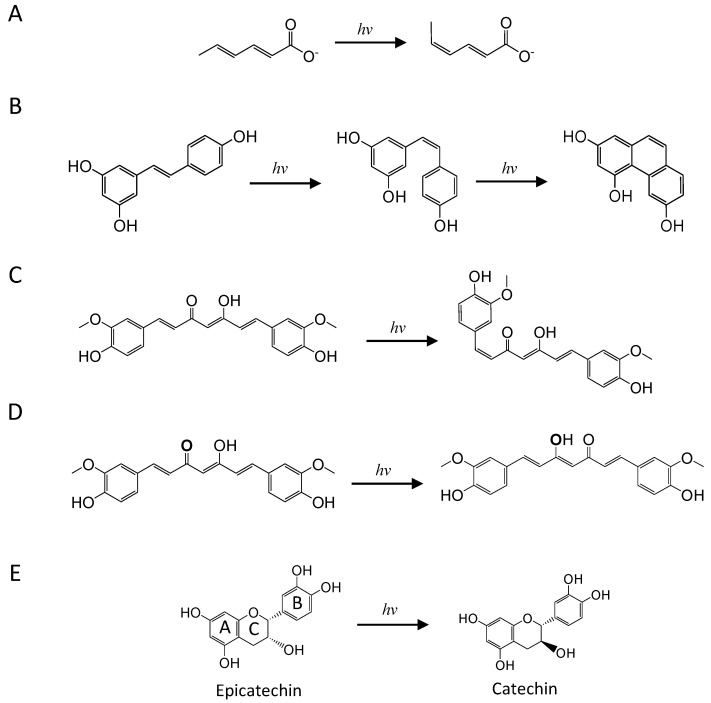
Isomerization, tautomerization, epimerization, and formation of new bonds to dissipate excitation energy in physical quenchers, from studies reported with UV-excited molecules. (**A**) Isomerization of *trans-* to *cis-* sorbate. (**B**) Isomerization of *trans-* to *cis-* resveratrol, and ring closure of *cis*- resveratrol to form the phenanthrene derivative. (**C**) Isomerization of *trans-* to *cis-* curcumin. The other two conjugated double bonds may also isomerize. (**D**) Tautomerization in the excited-state intramolecular proton transfer (ESIPT) reaction of curcumin. (**E**) Epimerization that converts epicatechin to catechin.

For conjugated double bonds in dienes such as sorbate, isomerization is a well-studied mode of triplet-energy dissipation [47,57] (Figure 5A) and *trans* to *cis* is the direction toward lower energy. The greater the number of double bonds, the greater the quenching rate constant [55]. Carotenoids, for example, are essentially sorbate with a longer conjugated double bond system and therefore accept and dissipate energy at lower energies [21]. 

Isomerization is also the mode of triplet-energy dissipation by the stilbenes, ring-containing conjugated systems including resveratrol [54]. There is evidence for photoinduced *trans*-*cis* isomerization of resveratrol. Moreover, once the *cis*-isomer is formed it is possible to form the phenanthrene-derivative by energy-consuming ring closure, a chemical-quenching mechanism (Figure 5B) [58,59,60]. The *cis*-isomers of a series of chlorine substituted stilbenes have been prepared by irradiating solutions of the *trans*-isomers [61]. Ferulic acid and curcumin similarly have a conjugated double bond exterior to a UV-absorbing aromatic ring, as does the less potent compound caffeic acid. Curcumin was the most efficient quencher of the triplet acetone formed by TMD cleavage. It has three *trans*-double bonds in its structure, suggesting than one or more can isomerize (in successive steps) (Figure 5C). Substantial amounts of the curcumin *cis*-isomer (named *trans*-enol) have been reported in polar protic solvents after UV irradiation [62]. To observe the *cis*-isomer, curcumin must be in the enol tautomer (wherein the *trans*-double bond lies between the two exocyclic oxygens) [62]. UVB irradiation of substituted α-arylcinnamates also causes isomerization. Because the double bonds involve substituents rather than H, the description is in terms of E- to Z- compounds rather than *trans* to *cis*, as well as the reverse direction [63]. This E-Z isomerization upon irradiation has also been observed on chloro- substituted α-arylcinnamates [64].

### 4.3. Triplet-Energy Dissipation by Tautomerization and Epimerization 

Additional radiation-less processes have been reported for the decay of the curcumin-excited state [62], including hydrogen bond vibration and both intramolecular and intermolecular proton exchange that interchanges keto and enol groups—a tautomerization reaction (Figure 5D). The intramolecular proton exchange, termed excited-state intramolecular proton transfer (ESIPT), requires electronic excitation and is favored in non-polar solvents where it is unperturbed by solvent-molecule interactions (hydrogen bonding or charge attraction), but is also possible in polar solvents such as water [62]. 

Epimerization requires breaking and re-forming a single bond and so dissipates energy. After exposure to UVC, epicatechin in Group II epimerizes to catechin due to a reorientation of the groups bound to the C ring of this flavonoid (Figure 5E) [65]. Conversion of epicatechin to catechin also occurs at pH > 6 at 40, 80, and 100 °C [66]. The epi → non-epi direction is observed due to the epi-isomer predominating in tea extract (~90%), as was used here, but the non-epi → epi conversion also occurs [66] and both have been reported for several catechins. The flavan structure of catechin is also present in epigallocatechin gallate (group III of Table 1), making it possible that the epimerization type of relaxation process also operates in that molecule.

### 4.4. Inhibitory Role of Glycosidic Sugars

In plants, glycosidic sugars are important for solubility and molecular transport. In the present biomolecules, we found that the glycosides polydatin, baicalin, and chlorogenic acid had a higher IC_50_ than their sugar-free analog. For polydatin and chlorogenic acid, the difference in triplet-quenching activity moved them into the next less-effective group. We had expected that polydatin would have an IC_50_ value similar to resveratrol because polydatin is a resveratrol molecule bound to a hexose; however, the IC_50_ value was six times greater. A similar phenomenon has been observed in the kinetics of quenching of triplet riboflavin by luteolin or quercetin versus rutin [67]; rutin contains a disaccharide moiety. Luteolin and quercetin quenched triplet riboflavin at 40–70% faster rates than rutin. It was hypothesized that the bulky sugar component of the molecule reduces the diffusion rate and increases the steric hinderance of rutin contacting triplet riboflavin [67]. 

### 4.5. Algal Compounds

Evaluating the trend of the algal extracts to quench triplet acetone using only three concentrations is just the starting point for a deeper study in which a few are subjected to purification, isolation, and identification of the active compounds, perhaps MAAs, for further analysis. Regarding the quenching effect of the MAA, exciting the MAA at the maximum absorption wavelength shows minimal fluorescence [36,39]. There is evidence of *cis-trans* isomerization (at low yield) from usujirene to palythine (see Figure 1) upon UVB exposure of the former [37]. The reverse reaction (palythine → usujirene) is 10 times less efficient [37]. In the case of palythine excitation and return to the ground state, the process occurs by a non-radiative mechanism. The excitation of palythine by triplet acetone causes the C=N bond of the molecule to rotate and releases the energy as heat [39].

### 4.6. Chemical Modes of Triplet-State Quenching

#### 4.6.1. Dioxetane Cleavage

**Figure 6:**
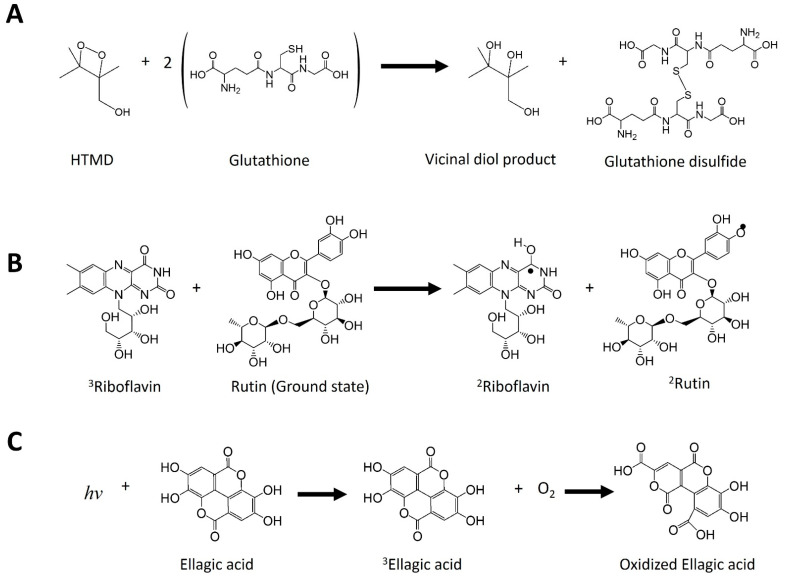
Chemically quenching of triplet-state energy by oxidation reactions of the second and third group compounds of Table 1. (**A**) Inactivation of 3-hydroxyethyl-3,4,4-trimethyl-1,2-dioextane (HTMD) by glutathione to form a ground-state vicinal diol molecule; (**B**) Electron transfer from a polyphenol to an excited triplet-state riboflavin, forming riboflavin and rutin radicals; (**C**) Ellagic acid that has physically quenched a triplet-state molecule by energy transfer is then physically quenched by O_2_ to create excited singlet oxygen, which is in turn chemically quenched by oxidizing another molecule of ellagic acid.

Thiols, as in glutathione, have been observed to “quench” triplet-state carbonyls by preventing them: cleaving the dioxetane O–O bond to diols, which are not excited to a triplet state by this level of energy (Figure 6A) [46,68]. Glutathione can also intercept a superoxide (or peroxyl) radical before a dioxetane can form [68]. The latter reaction with glutathione unfortunately creates ^1^O_2_ [68].

#### 4.6.2. Ground-State Electron Transfer

Energy transfer from triplet states via Dexter electron exchange can, in principle, be blocked by the typical activity of antioxidants—ground-state electron transfer. This activity is the transfer of one electron from the quencher to the excited molecule, resembling half of a Dexter exchange (Figure 6B). A model for the flavonoids is provided by the observation that triplet riboflavin (^3^Rbf) can be deactivated by the polyphenols rutin, catechin, and epigallocatechin gallate in a concentration-dependent manner [69]. The triplet deactivation was by transfer from the polyphenol to ^3^Rbf of either a hydrogen atom (H^•^, i.e., an electron accompanied by a proton) or just the electron. This ground-state transfer produces a polyphenol radical and a (doublet) riboflavin radical (^2^Rbf, i.e., the radical ^2^Rbf^•^) [67,69]. Filling the ground-state vacancy left by the riboflavin’s excited electron may prevent Dexter exchange, blocking that mode of energy transfer to DNA and thereby preventing dCPD formation. The high energy electron remains on the riboflavin, which can still be considered excited. Doublet riboflavin (or in our case DBAS^•^) can then donate the extra electron (and proton) to an electron acceptor, which in the case of ambient O_2_ would result in the reactive oxygen species superoxide, O_2_^•−^ [69]. These ground-state transfer reactions are also not a desirable property for a triplet-state.

#### 4.6.3. Triplet-Energy Dissipation to Ambient Oxygen

The distinction between physical and chemical triplet quenchers is not always tidy. Ellagic acid in Group II differs from the other triplet quenchers here analyzed. Because of the absence of *trans*-double bonds, an isomerization reaction does not occur. Its planarity [70] prevents an epimerization reaction. However, when an aerated solution of ellagic acid is UV irradiated, it is excited to a singlet state. It then intersystem crosses to a triplet state and transfers this energy to ambient ^3^O_2_ to generate singlet oxygen, ^1^O_2_, an excited state of ^3^O_2_. We presume that TMD excites ellagic acid to the triplet state directly. Thus far, we have two physical triplet energy transfers. However, the ^1^O_2_ then adds to another molecule of ellagic acid, creating a dioxetane that is unstable; its cleavage opens an aromatic ring and leads to subsequent rearrangements (Figure 6C). The resulting oxidized ellagic acid was confirmed by spectroscopic techniques and by density functional theory (DFT) [71]. Triplet-energy dissipation to O_2_, making deleterious ^1^O_2_, is clearly not a desirable property for a triplet-state quencher. It is clearly important to understand the mechanism of a quencher’s action before using it in an application.

### 4.7. Roles of Physical and Chemical Quenching

The striking ‘isomerizability’ of the Group I ‘hits’ suggests that this energy-intensive process is dissipating transferred energy. Therefore, these compounds appear to quench triplet states by diverting Dexter energy transfer, rather than by the usual antioxidant activity of donating ground-state electrons that cause nonproductive cleavage of dioxetanes, enhance intersystem crossing, or block Dexter electron exchange. For Groups II and III, however, chemical quenching of triplet states by electron donation is more likely. Direct confirmation of specific mechanisms of triplet-quenching and energy dissipation by these antioxidants will require HPLC, NMR, and EPR characterization to detect isomerization products and radical intermediates. 

### 4.8. Skin Delivery

Topical delivery of molecules into skin requires that they be nonpolar to penetrate the stratum corneum and polar to reach the living keratinocytes and melanocytes [72]. Delivery strategies are therefore largely empirical and non-optimal. Ferulic acid delivered topically reaches a concentration of only 25 pM in 48 h [73], whereas topical ascorbic acid reaches 1 mM after a single application [74]. It is therefore fortunate that quenching of dCPDs occurred well below the IC_50_ for quenching of DBAS luminescence (Table 4). Delivering triplet quenchers topically may require optimizing analogs or using biocompatible lipophilic nanoparticles as carriers to traverse the stratum corneum. Higher concentrations of triplet quenchers are reached with dietary supplementation. Single doses of curcumin, resveratrol, or epigallocatechin gallate reach 0.3–7 μM [75,76,77], so repeated dietary supplementation would be more likely to attain their DBAS IC_50_. Vitamin E supplementation for one month reaches 27 mM [78], well above its 427 μM IC_50_. Cytosolic glutathione is 11 mM even without supplementation [79]. The latter two molecules decline precipitously after UV exposure, however, so it has been proposed that augmentation will be beneficial [80].

## 5. Conclusions

These experiments yielded four results: (i) Chemiexcitation of the triplet-state reporter DBAS can be impeded or prevented by using natural products. Many of the effective compounds were already known as antioxidants; (ii) Biomolecules selected on the basis of having conjugated double bonds providing delocalized electrons were particularly effective at diverting energy from triplet-state excited molecules and not allowing the DBAS reporter to emit the energy as light; (iii) Compounds fell into three groups according to their effectiveness in quenching the triplet state, with the most effective being active in the 50 μM range. Comparison to the literature suggests different chemical mechanisms operative in each group and (iv) This excited-state quenching activity also prevented the formation of the mutagenic and lethal CPD in DNA in vitro and in cells. This understanding may provide the basis for new approaches to protecting skin, as the most effective quenchers appear to act by energy transfer rather than the electron-donation mechanism for which common antioxidants are best known.

## Figures and Tables

**Table 2:** Concentration range and IC_50_ value of a natural product mixture that quenches triplet-state energy, assessed as TMD-DBAS luminescence.

Mixture	Luminescence Quenching Range (μg/mL)	IC_50_ Value (μg/mL)
Potassium Sorbate		8.7
Chlorogenic acid	0.018–5315	195
Honeysuckle Extract (neochlorogenic acid, chlorogenic acid, isochlorogenic acid A, rutin, quercetin, and cyanidin-3-glucoside)	0.03–9000	245 ± 25
Epigallocatechin Gallate		320

**Table 3:** Percentage inhibition of TMD-DBAS luminescence by the most effective algae extracts.

Natural Products ID	Harvest Site	Genus	Species	Extract Type	% Inhibition at 50 μg/mL	% Inhibition at 500 μg/mL
J003678	Mauritius	*Acanthophora*	*muscoides*	Aqueous	35	90
J004770	Florida	*Gracilaria*	*vermiculophylla*	Aqueous	73	58
J001263	Southern New Zealand	*Porphyra*		Organic	8	74
J002656		*Acanthophora*	*muscoides*	Aqueous	24	72
J003392	Tanzania (Tanganyika, Zanzibar)	*Gracilaria*		Aqueous	24	44
J004034	Florida	*Gracilaria*	*foliifera*	Aqueous	56	64

**Table 4:** Percentage of dCPD inhibition upon addition of 10 μM of triplet-state quencher to dT_150_ or ^CT^DNA samples treated with TMD. For comparison, the middle column shows the ratio of the quencher concentration to its IC_50_ in the reporter assay.

Group	Compound	Conc/IC_50_	% dCPD Inhibition	
			dT_150_	^CT^DNA
I	Potassium Sorbate	0.2	58 ± 10%	74 ± 7%
II	Epicatechin	0.04	71 ± 20%	41 ± 6%
III	Luteolin	0.02	90 ± 12%	40 ± 1%

## Data Availability

All data are presented in the text or Supplemental Material.

## Supplementary Materials

The following are available online at https://www.mdpi.com/article/10.3390/antiox11020357/s1, Supplementary Methods. Supplementary Data Figure S1: Chemiluminescence of TMD-DBAS excitation in presence of curcumin, Figure S2: resveratrol, Figure S3: potassium sorbate, Figure S4: ferulic acid, Figure S5: baicalein, Figure S6: polydatin, Figure S7: glutathione, Figure S8: baicalin, Figure S9: caffeic acid, Figure S10: epicatechin, Figure S11: myricetin, Figure S12: ellagic acid, Figure S13: Trolox, Figure S14: chlorogenic acid, Figure S15: luteolin, Figure S16: epigallocatechin gallate, Figure S17: gallic acid, Figure S18: honeysuckle extract, Figure S19: algae extracts, Figure S20: dCPD quenching activity before and after correcting for ELISA assay nonlinearity by using the UVC calibration curve. Table S1: Triplet-state quenching survey, Table S2: TMD-DBAS quenching activity of algal extracts. References [81,82,83,84] are cited in the supplementary materials.
