# Supplementary material for: Triplet-Energy Quenching Functions of Antioxidant Molecules
Source: Antioxidants (Basel). 2022 Feb 11;11(2):357. doi: 10.3390/antiox11020357 (PMC8868474; doi:10.3390/antiox11020357)
Supplement: Supplementary file 1 [file antioxidants-11-00357-s001.zip › antioxidants-1520830-supplementary data.pdf]

SUPPLEMENTARY DATA

Triplet-Energy Quenching Functions of Antioxidant Biomolecules

Carlos Angelé-Martínez, Leticia C. P. Goncalves, Sanjay Premi, Felipe A. Augusto, Meg A. Palmatier, Saroj K. Amar, and Douglas E. Brash

TABLE OF CONTENTS

Figure S1. Chemiluminescence of TMD-DBAS excitation in the presence of **curcumin**. 2

Figure S2. Chemiluminescence of TMD-DBAS triplet excitation in the presence of **resveratrol**. 3

Figure S3. Chemiluminescence of TMD-DBAS excitation in the presence of **potassium sorbate**. 4

Figure S4. Chemiluminescence of TMD-DBAS triplet excitation in the presence of **ferulic acid**. 5

Figure S5. Chemiluminescence of TMD-DBAS excitation in the presence of **baicalein**. 6

Figure S6. Chemiluminescence of TMD-DBAS excitation in the presence of **polydatin**. 7

Figure S7. Chemiluminescence of TMD-DBAS excitation in the presence of **glutathione**. 8

Figure S8. Chemiluminescence of TMD-DBAS excitation in the presence of **baicalin**. 9

Figure S9. Chemiluminescence of TMD-DBAS excitation in the presence of **caffeic acid**. 10

Figure S10. Chemiluminescence of TMD-DBAS excitation in the presence of **epicatechin**. 11

Figure S11. Chemiluminescence of TMD-DBAS excitation in the presence of **myricetin**. 12

Figure S12. Chemiluminescence from TMD-DBAS triplet excitation in the presence of **ellagic acid**. 13

Figure S13. Chemiluminescence from TMD-DBAS triplet excitation in the presence of **Trolox**. 14

Figure S14. Chemiluminescence from TMD-DBAS triplet excitation in the presence of **chlorogenic acid**. 15

Figure S15. Chemiluminescence of TMD-DBAS excitation in the presence of **luteolin**. 16

Figure S16. Chemiluminescence of TMD-DBAS excitation in the presence of **epigallocatechin gallate**. 17

Figure S17. Chemiluminescence of TMD-DBAS excitation in the presence of **gallic acid**. 18

Figure S18. Chemiluminescence of TMD-DBAS triplet excitation in the presence of **honeysuckle extract**. 19

Figure S19. Chemiluminescence of TMD-DBAS triplet excitation in the presence of **algae extracts** 20

Figure S20. dCPD quenching activity before and after correcting for ELISA assay nonlinearity 22

Table S1. Triplet-state quenching survey. 23

Table S2. TMD-DBAS quenching activity of algal extracts. 24

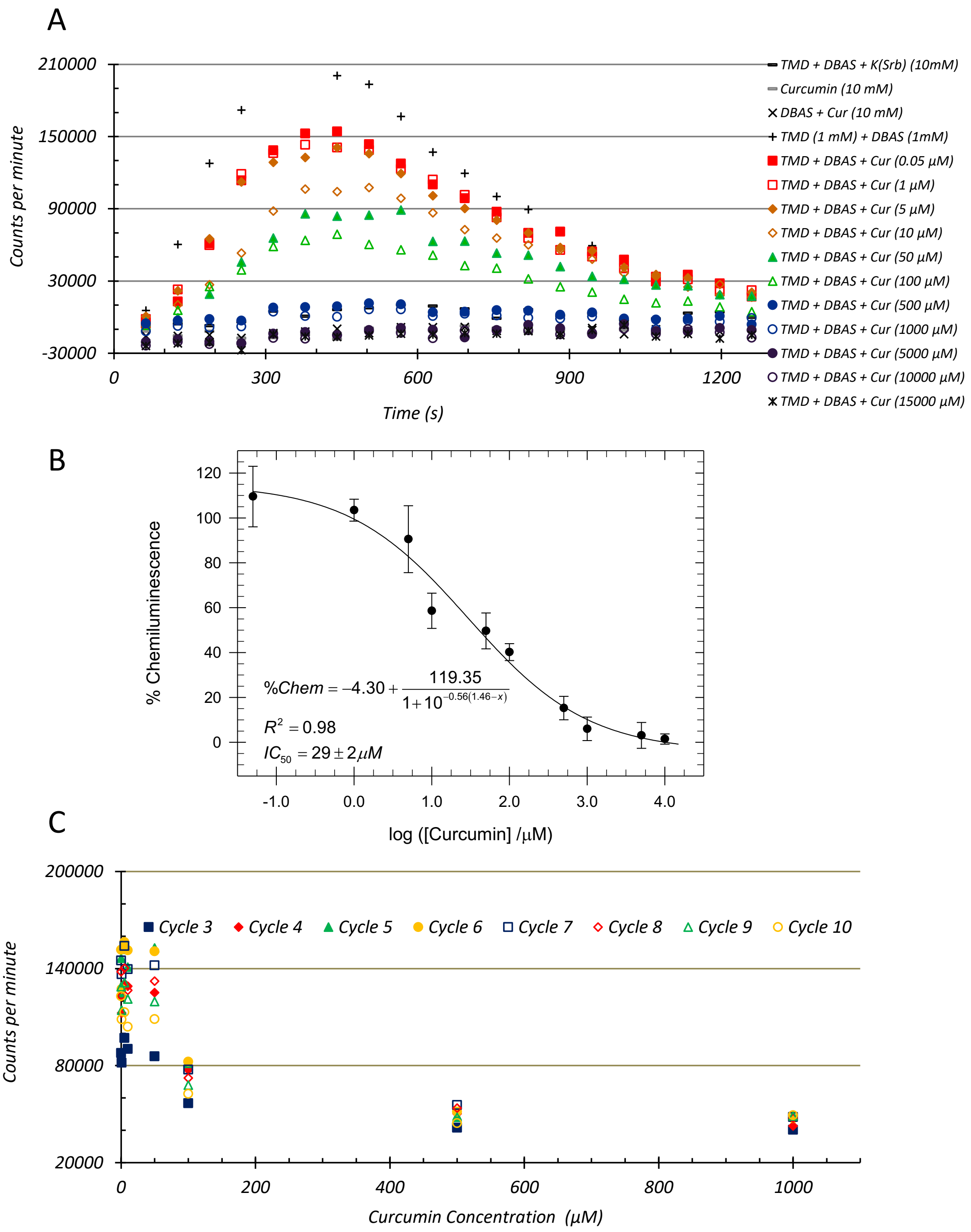

Figure S1. Chemiluminescence of TMD-DBAS excitation in the presence of **curcumin**.

(A) TMD-DBAS chemiluminescence with respect to time at different concentrations of curcumin.

(B) IC_{50} curve for the percentage reduction of chemiluminescence of TMD-DBAS excitation vs logarithm of concentration of curcumin.

(C) TMD-DBAS chemiluminescence with respect to concentration of curcumin at different cycles of measurement (1 cycle = 63 s).

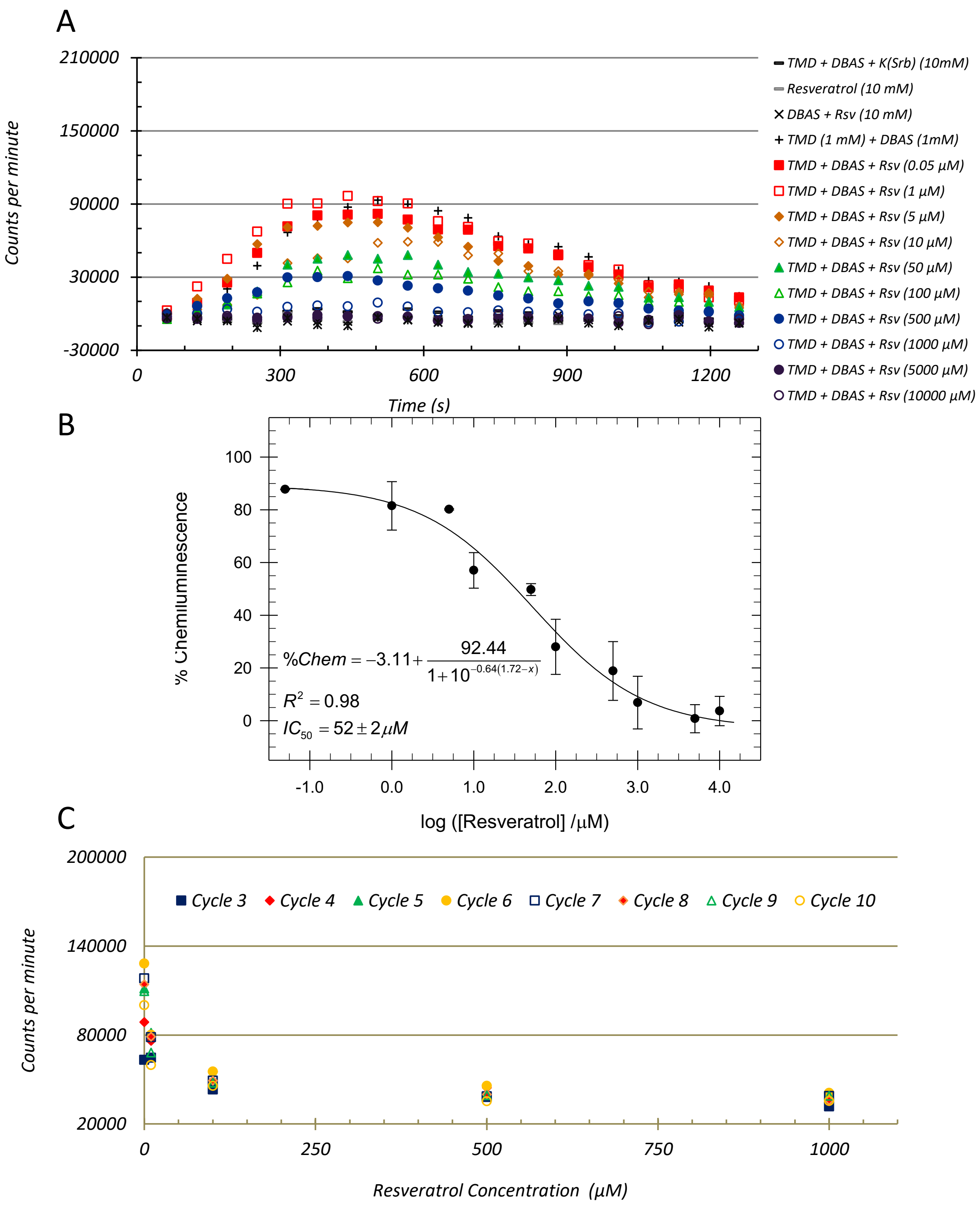

Figure S2. Chemiluminescence of TMD-DBAS triplet excitation in the presence of **resveratrol**.

(A) TMD-DBAS chemiluminescence with respect to time at different concentrations of resveratrol.

(B) IC_{50} curve for the percentage reduction of chemiluminescence of TMD-DBAS excitation vs logarithm of concentration of resveratrol.

(C) TMD-DBAS chemiluminescence with respect to concentration of resveratrol at different cycles of measurement (1 cycle = 63 s).

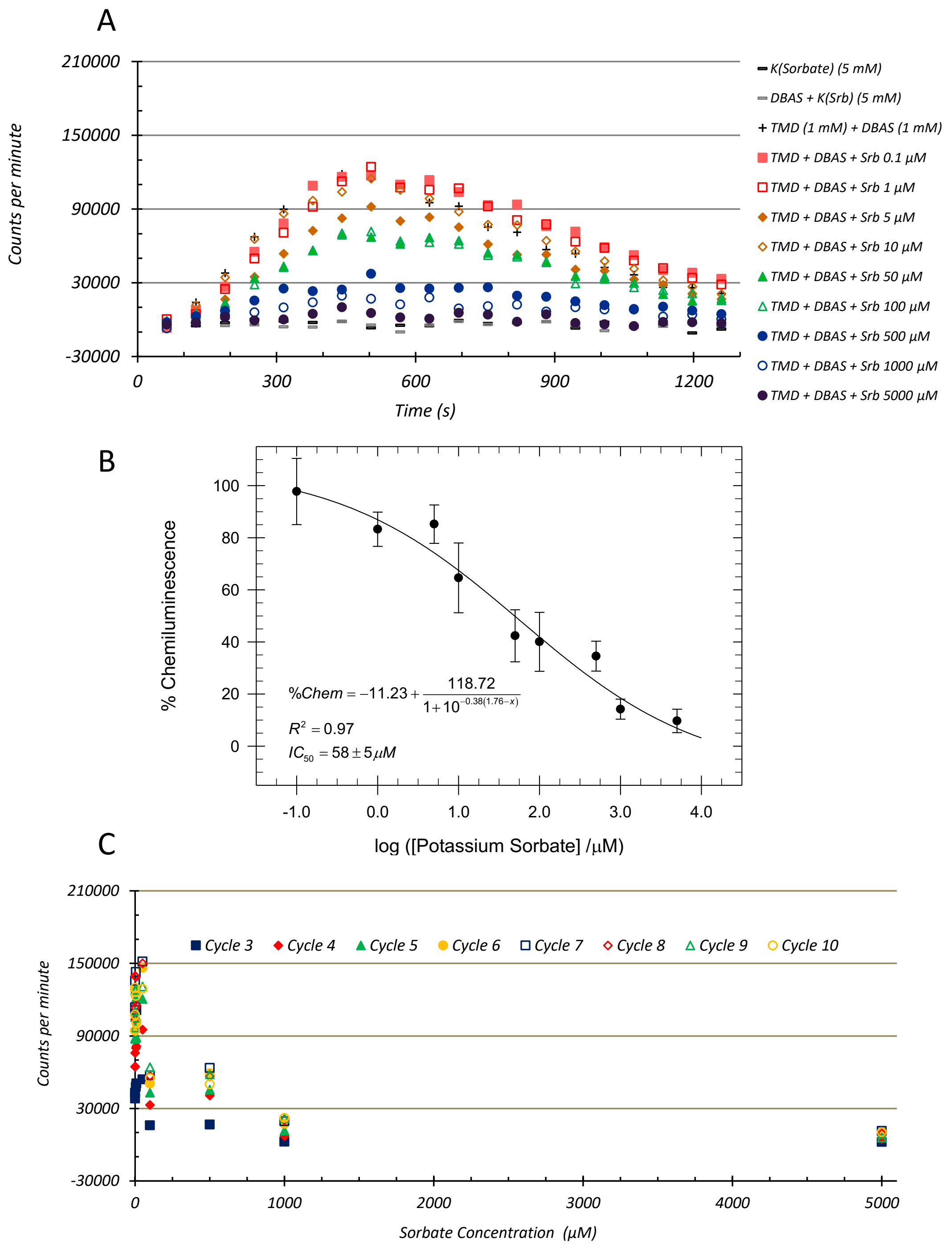

Figure S3. Chemiluminescence of TMD-DBAS excitation in the presence of **potassium sorbate**.

(A) TMD-DBAS chemiluminescence with respect to time at different concentrations of potassium sorbate (Srb).

(B) IC_{50} curve for the percentage of reduction of chemiluminescence of TMD-DBAS excitation vs logarithm of concentration potassium sorbate.

(C) TMD-DBAS chemiluminescence with respect to concentration of potassium sorbate at different cycles of measurement (1 cycle = 63 s).

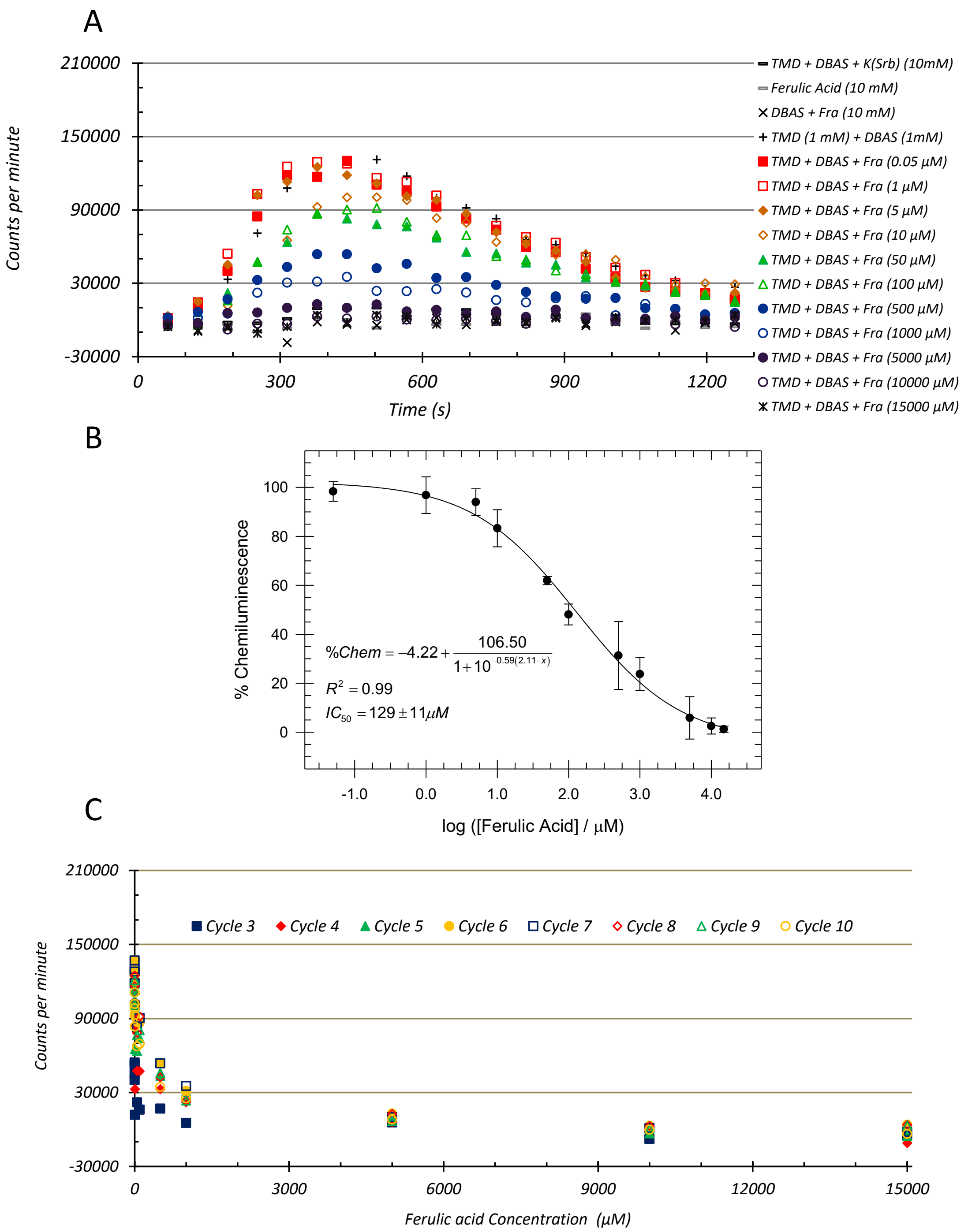

Figure S4. Chemiluminescence of TMD-DBAS triplet excitation in the presence of **ferulic acid**.

(A) TMD-DBAS chemiluminescence with respect to time at different concentrations of ferulic acid.

(B) IC_{50} curve for the percentage of reduction of chemiluminescence of TMD-DBAS excitation vs logarithm of concentration of ferulic acid.

(C) TMD-DBAS chemiluminescence with respect to concentration of ferulic acid at different cycles of measurement (1 cycle = 63 s).

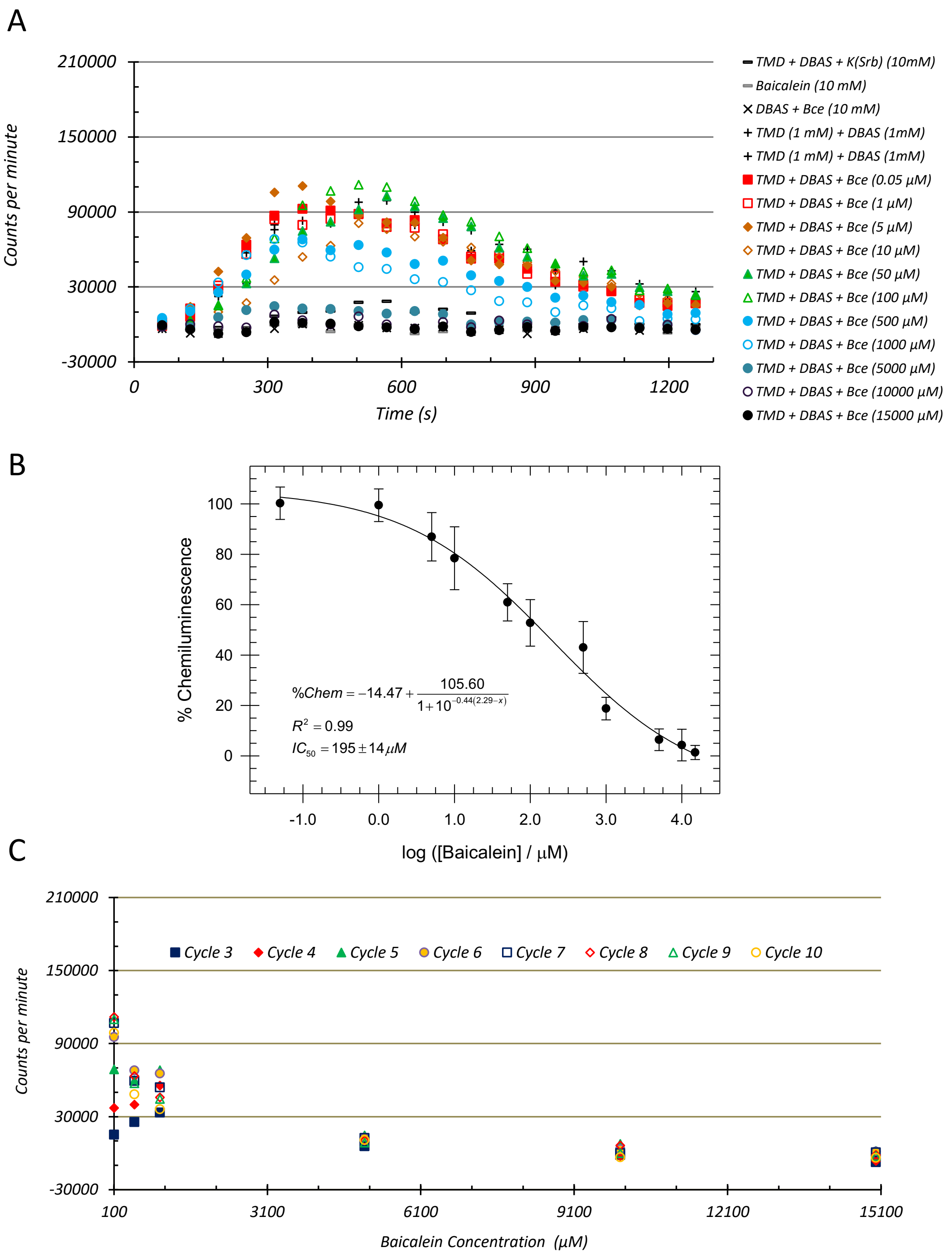

Figure S5. Chemiluminescence of TMD-DBAS excitation in the presence of **baicalein**.

(A) TMD-DBAS chemiluminescence with respect to time at different concentrations of baicalein.

(B) IC_{50} curve for the percentage of reduction of chemiluminescence of TMD-DBAS excitation vs logarithm of concentration baicalein.

(C) TMD-DBAS chemiluminescence with respect to concentration of baicalein at different cycles of measurement (1 cycle = 63 s).

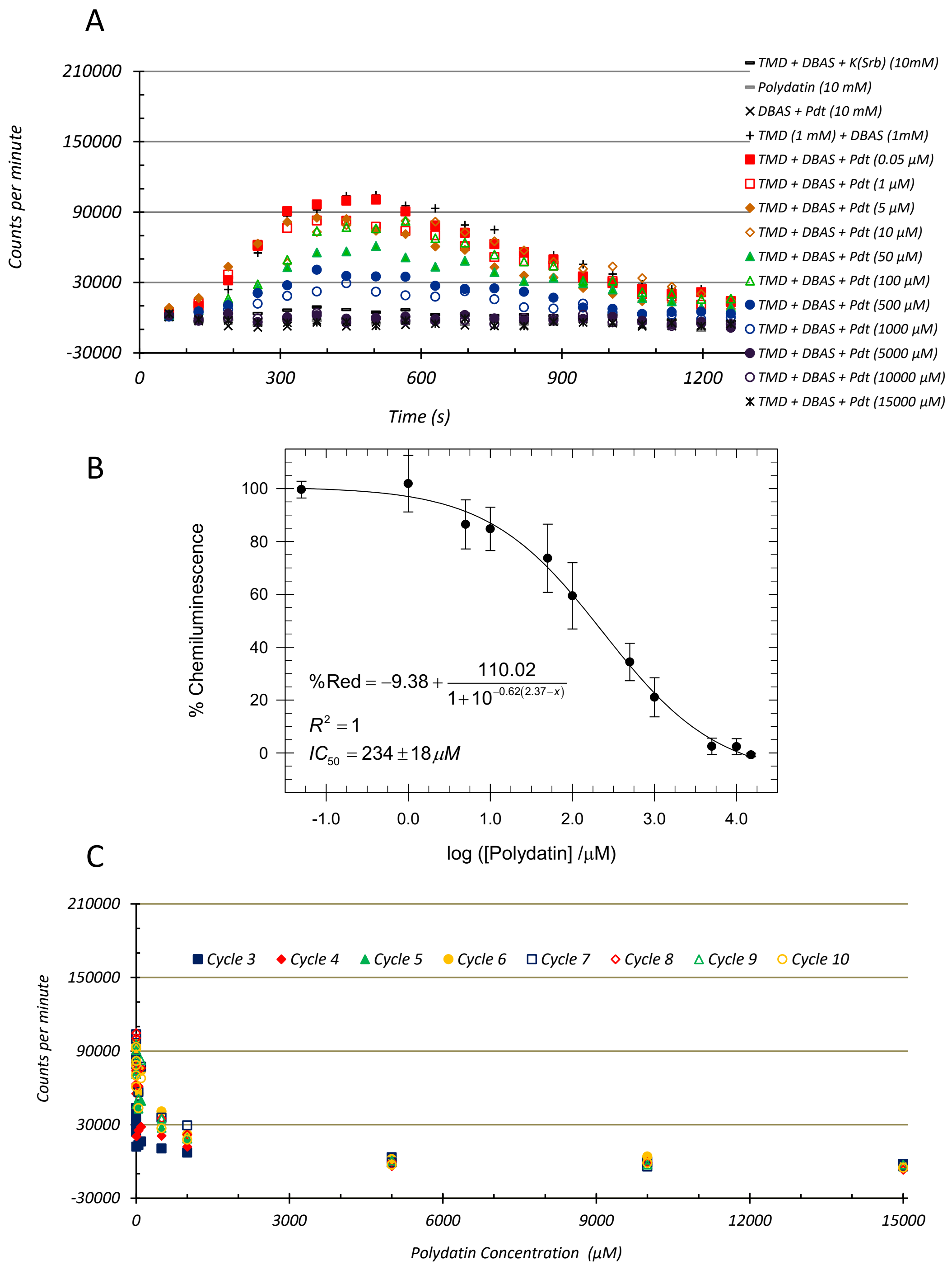

Figure S6. Chemiluminescence of TMD-DBAS excitation in the presence of **polydatin**.

(A) TMD-DBAS chemiluminescence with respect to time at different concentrations of polydatin.

(B) IC_{50} curve for the percentage of reduction of chemiluminescence of TMD-DBAS excitation vs logarithm of concentration polydatin.

(C) TMD-DBAS chemiluminescence with respect to concentration of polydatin at different cycles of measurement (1 cycle = 63 s).

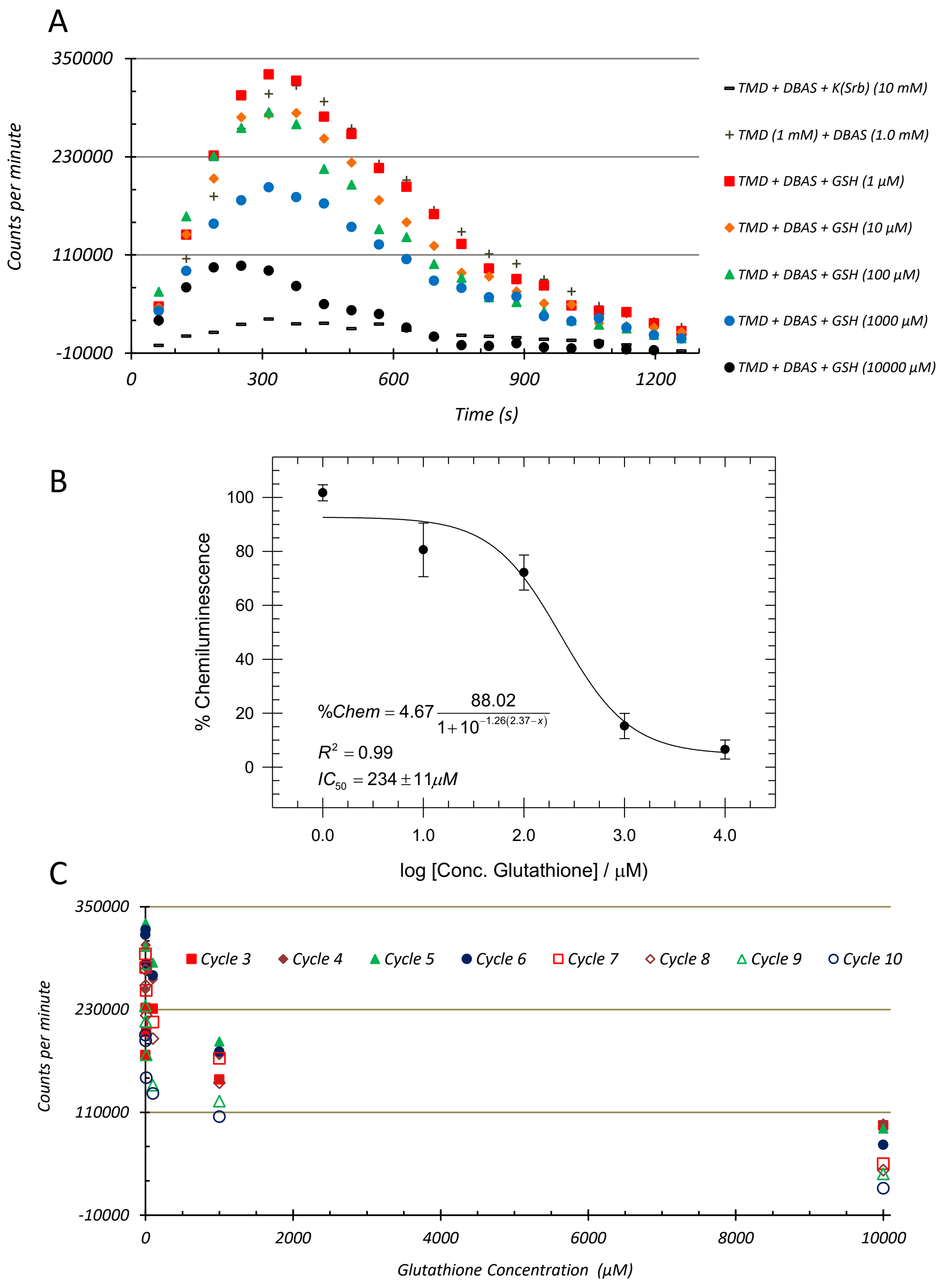

Figure S7. Chemiluminescence of TMD-DBAS excitation in the presence of **glutathione**.

(A) TMD-DBAS chemiluminescence with respect to time at different concentrations of glutathione.

(B) IC_{50} curve for the percentage of reduction of chemiluminescence of TMD-DBAS excitation vs logarithm of concentration glutathione.

(C) TMD-DBAS chemiluminescence with respect to concentration of glutathione at different cycles of measurement (1 cycle = 63 s).

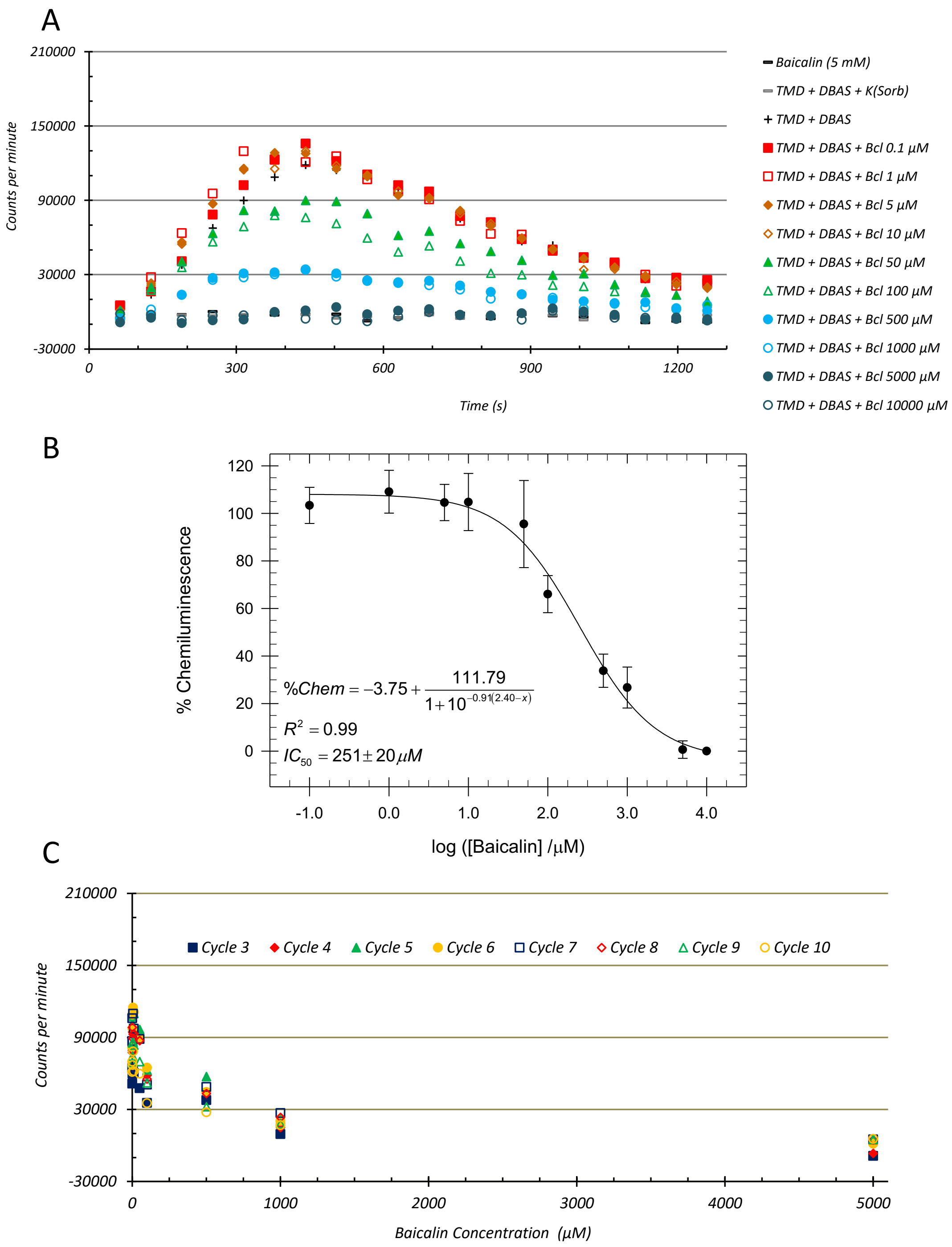

Figure S8. Chemiluminescence of TMD-DBAS excitation in the presence of **baicalin**.

(A) TMD-DBAS chemiluminescence with respect to time at different concentrations of baicalin.

(B) IC_{50} curve for the percentage of reduction of chemiluminescence of TMD-DBAS excitation vs logarithm of concentration of baicalin.

(C) TMD-DBAS chemiluminescence with respect to concentration of baicalin at different cycles of measurement (1 cycle = 63 s).

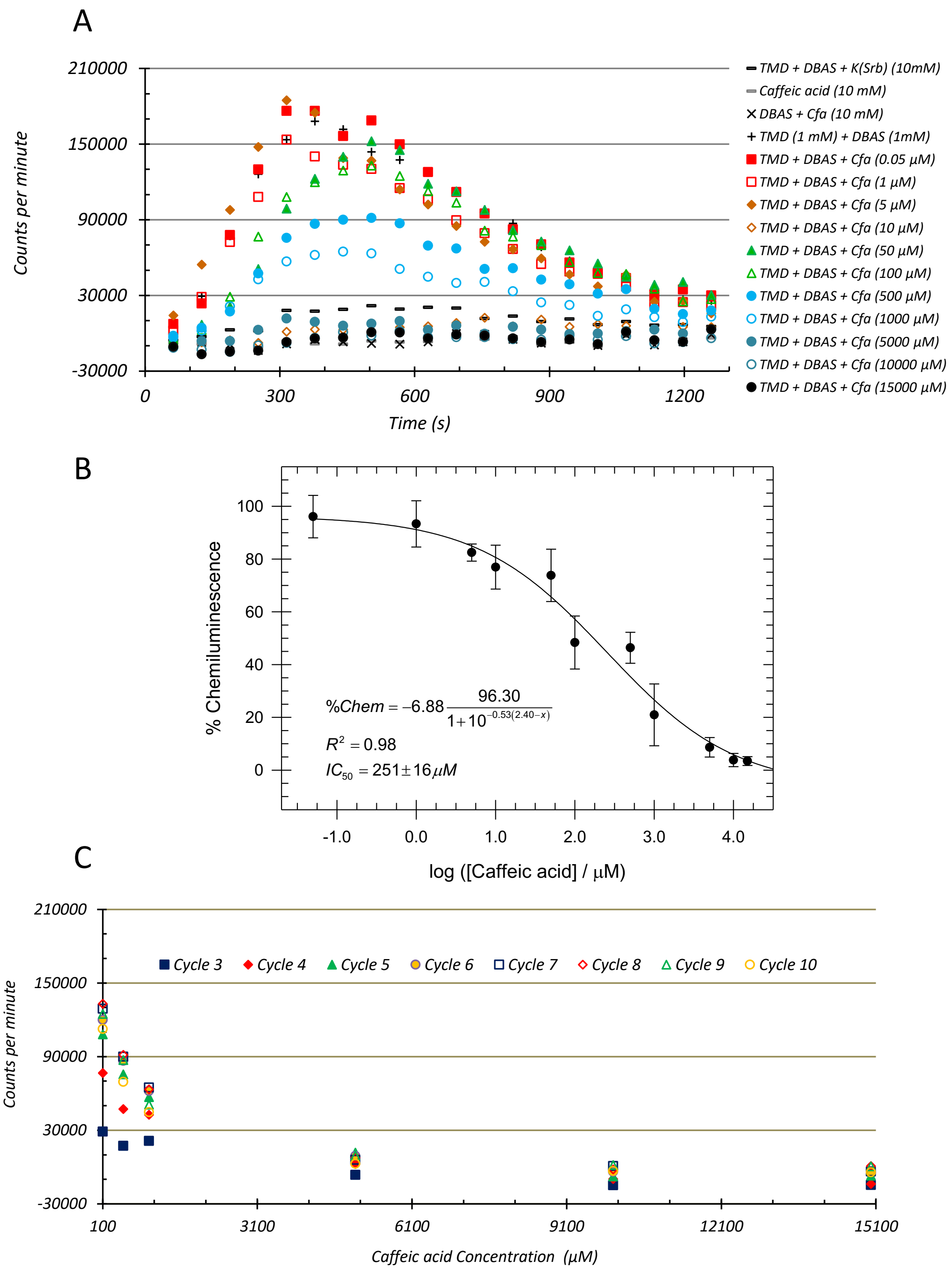

Figure S9. Chemiluminescence of TMD-DBAS excitation in the presence of **caffeic acid**.

(A) TMD-DBAS chemiluminescence with respect to time at different concentrations of caffeic acid.

(B) IC_{50} curve for the percentage of reduction of chemiluminescence of TMD-DBAS excitation vs logarithm of concentration of caffeic acid.

(C) TMD-DBAS chemiluminescence with respect to concentration of caffeic acid at different cycles of measurement (1 cycle = 63 s).

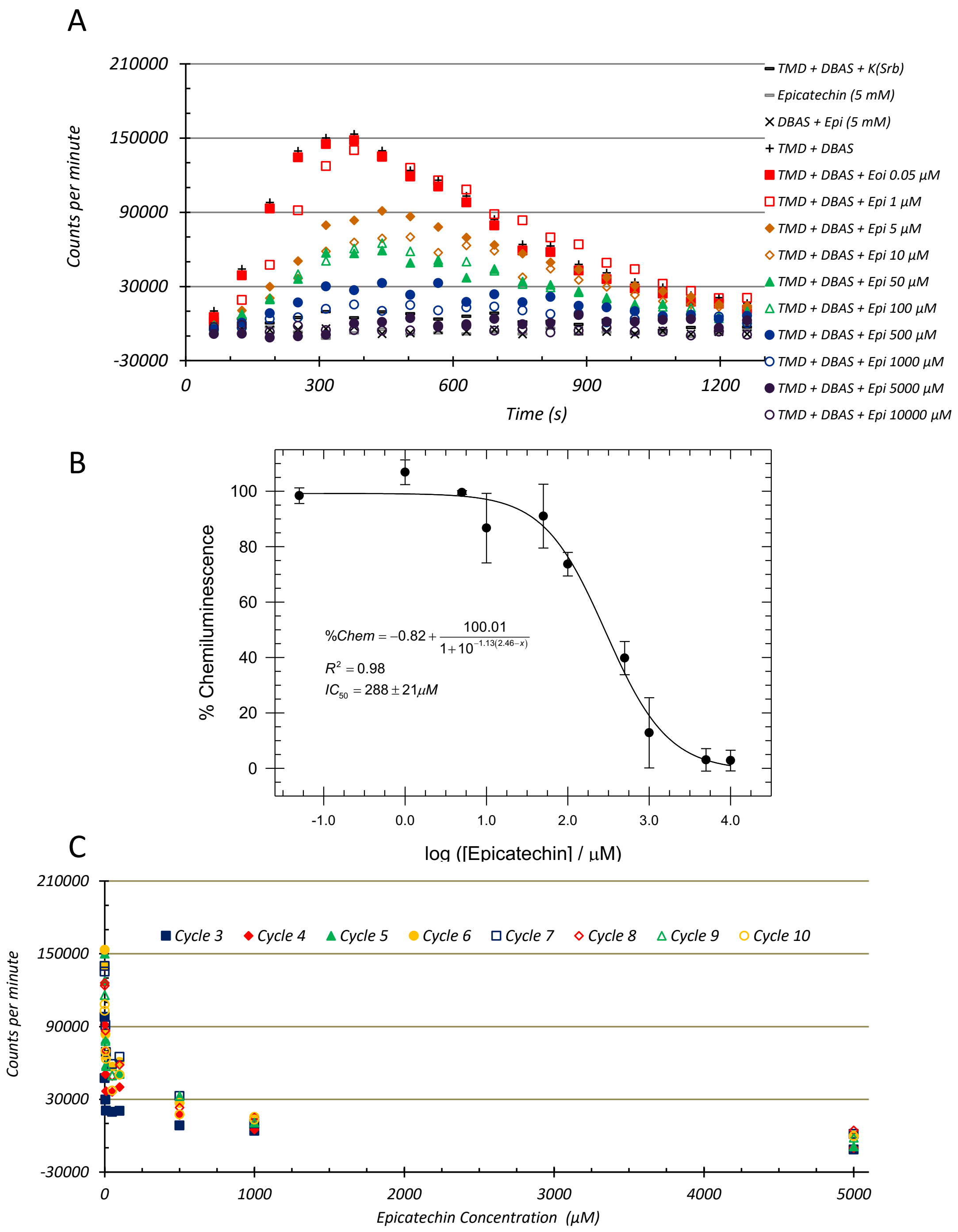

Figure S10. Chemiluminescence of TMD-DBAS excitation in the presence of **epicatechin**.

(A) TMD-DBAS chemiluminescence with respect to time at different concentrations of catechin.

(B) IC_{50} curve for the percentage of reduction of chemiluminescence of TMD-DBAS excitation vs logarithm of concentration catechin.

(C) TMD-DBAS chemiluminescence with respect to concentration of catechin at different cycles of measurement (1 cycle = 63 s).

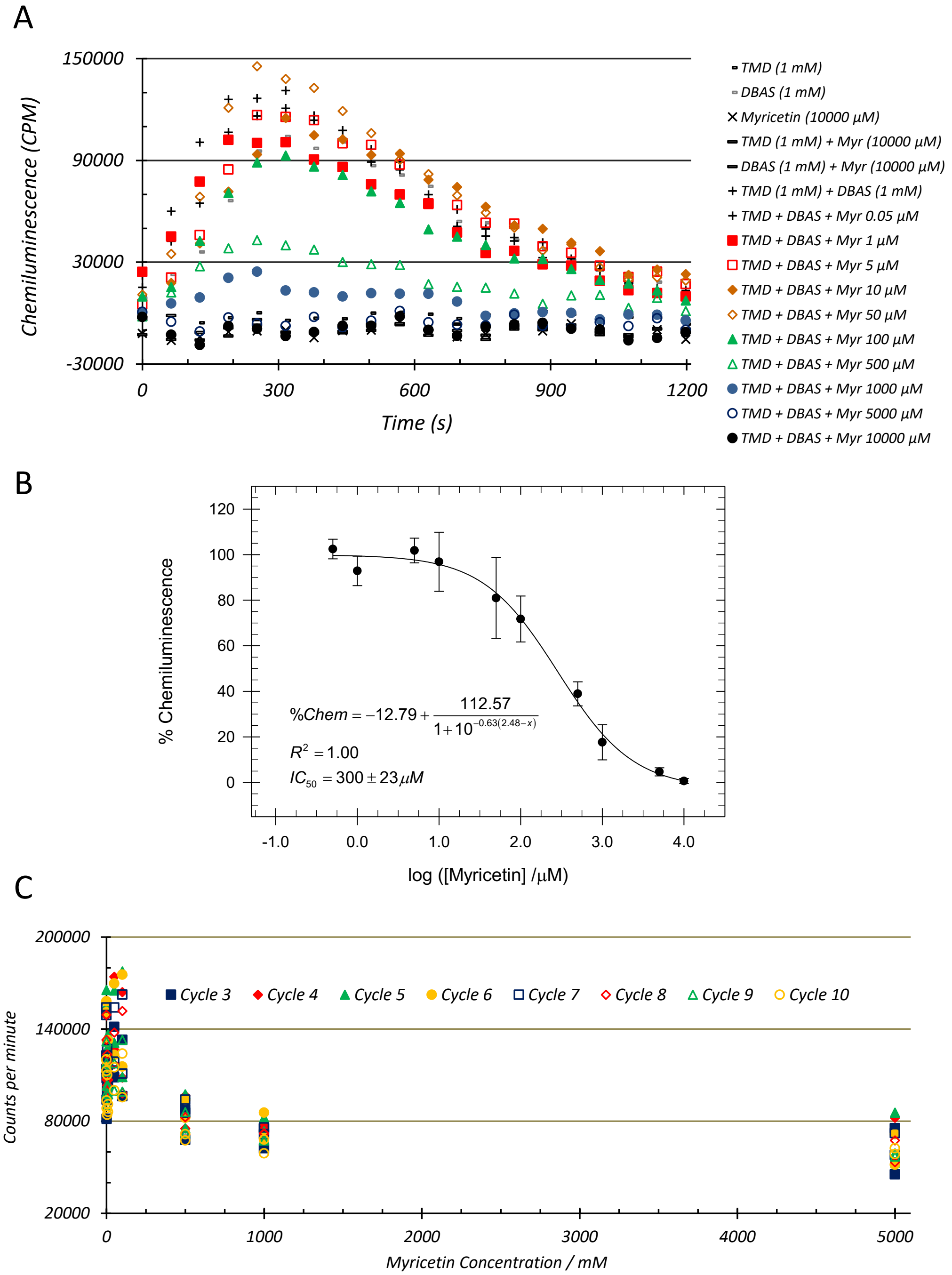

Figure S11. Chemiluminescence of TMD-DBAS excitation in the presence of **myricetin**.

(A) TMD-DBAS chemiluminescence with respect to time at different concentrations of myricetin.

(B) IC_{50} curve for the percentage of reduction of chemiluminescence of TMD-DBAS excitation vs logarithm of concentration of myricetin.

(C) TMD-DBAS chemiluminescence with respect to concentration of myricetin at different cycles of measurement (1 cycle = 63 s).

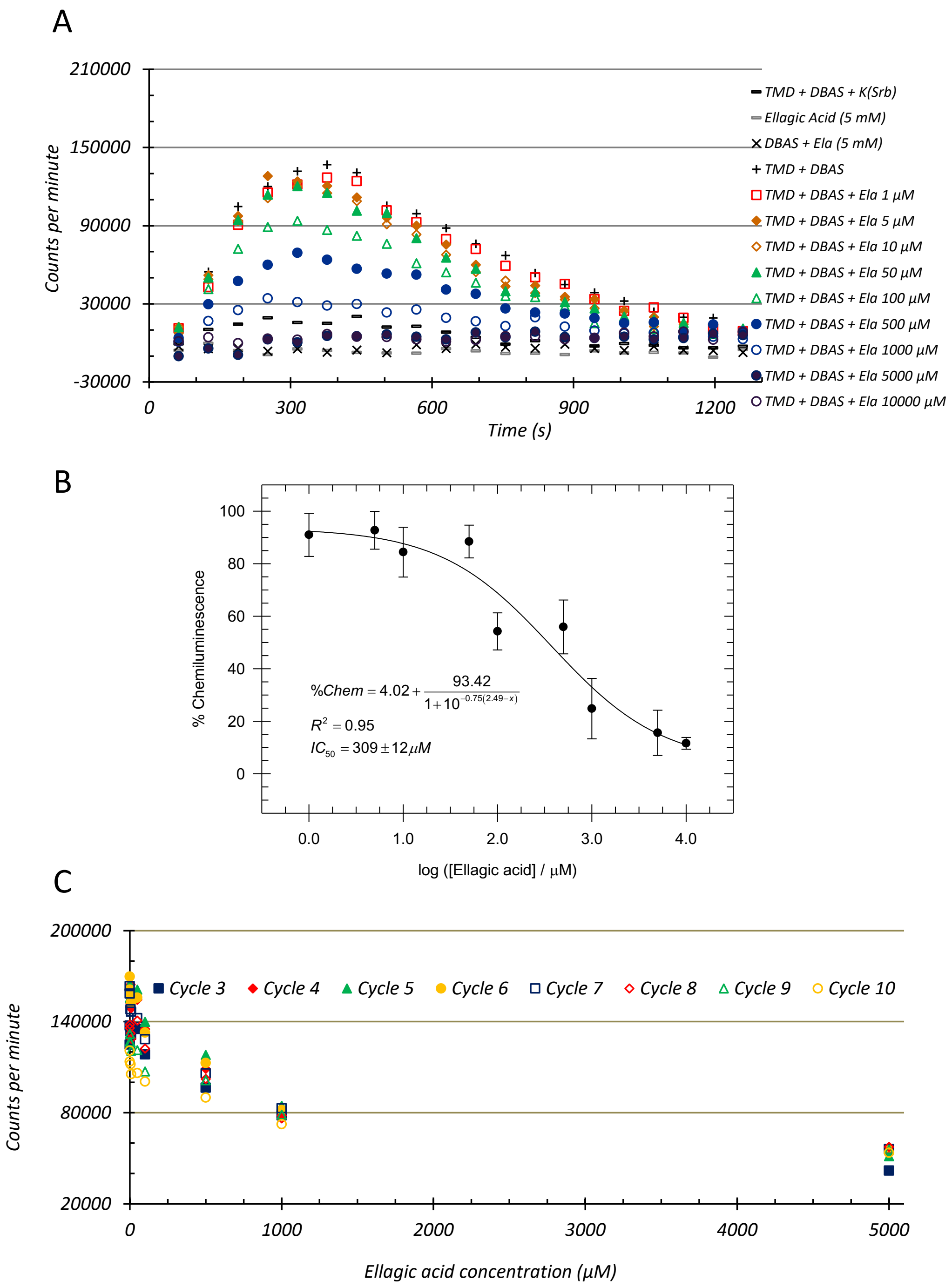

Figure S12. Chemiluminescence from TMD-DBAS triplet excitation in the presence of **ellagic acid**.
(A) TMD-DBAS chemiluminescence with respect to time at different concentrations of ellagic acid.
(B) IC_{50} curve for the percentage of reduction of chemiluminescence of TMD-DBAS excitation vs logarithm of concentration of ellagic acid.
(C) TMD-DBAS chemiluminescence with respect to concentration of ellagic acid at different cycles of measurement (1 cycle = 63 s).

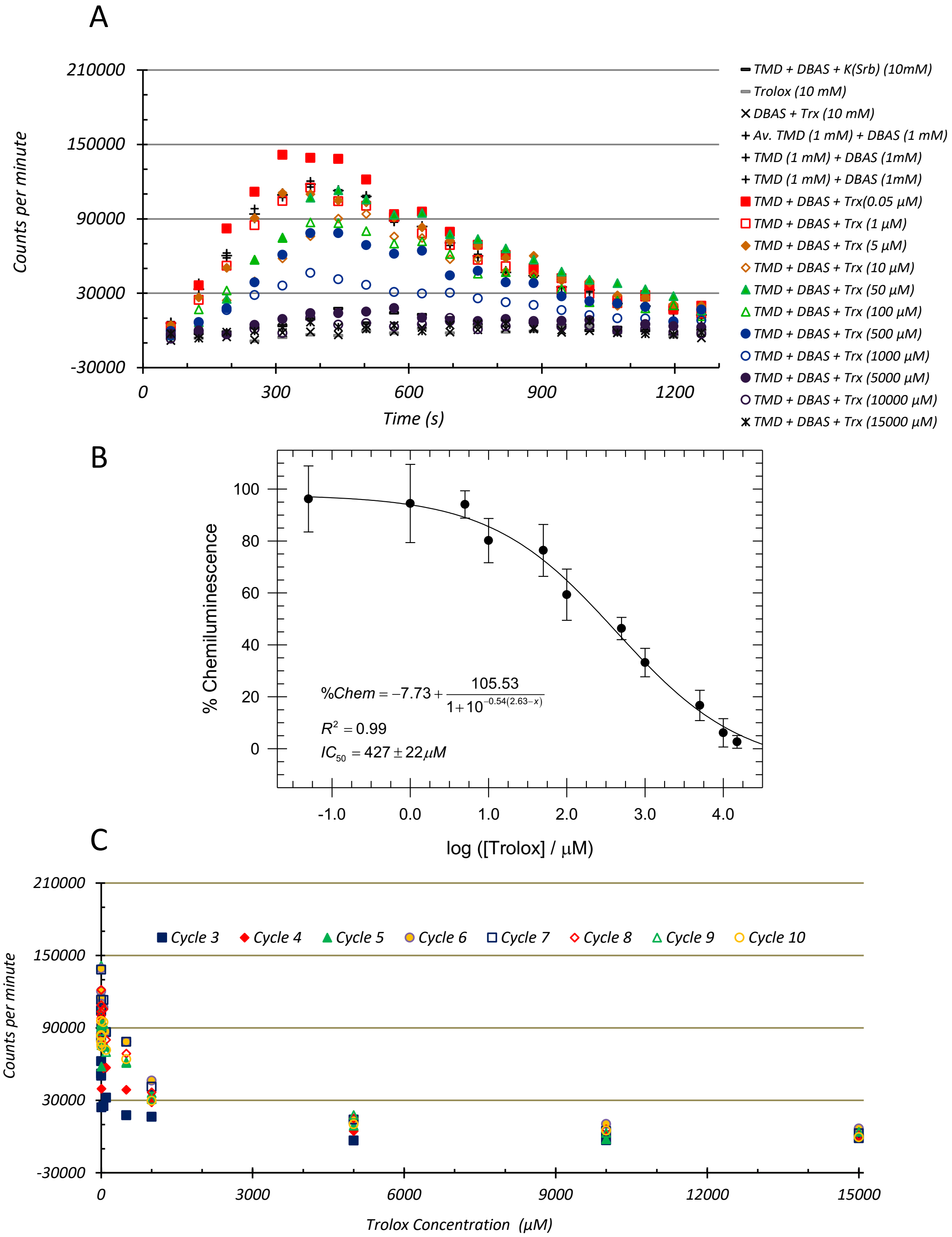

Figure S13. Chemiluminescence from TMD-DBAS triplet excitation in the presence of **Trolox**.

(A) TMD-DBAS chemiluminescence with respect to time at different concentrations of Trolox.

(B) IC_{50} curve for the percentage of reduction of chemiluminescence of TMD-DBAS excitation vs logarithm of concentration of Trolox.

(C) TMD-DBAS chemiluminescence with respect to concentration of Trolox at different cycles of measurement (1 cycle = 63 s).

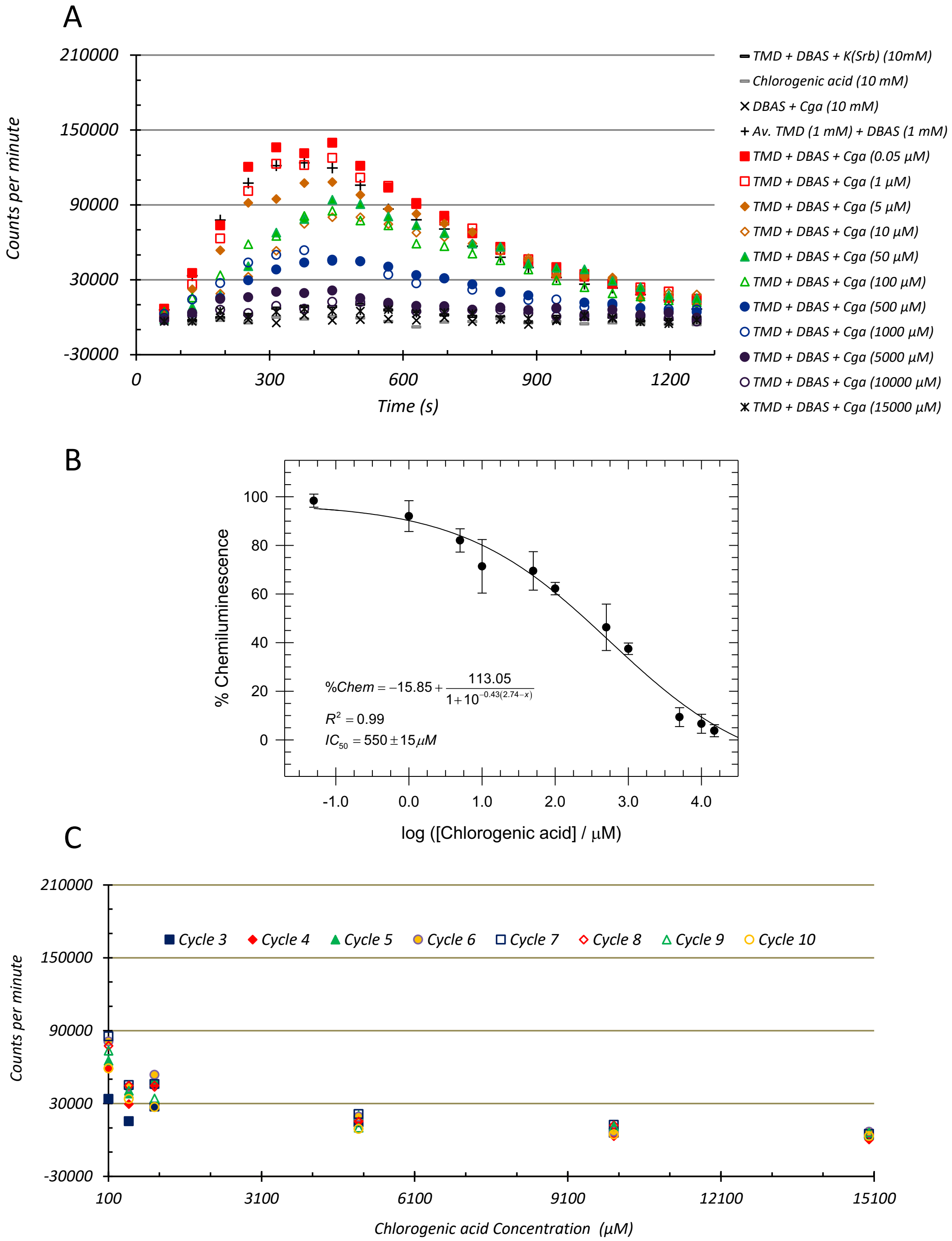

Figure S14. Chemiluminescence from TMD-DBAS triplet excitation in the presence of **chlorogenic acid**.

(A) TMD-DBAS chemiluminescence with respect to time at different concentrations of chlorogenic acid.

(B) IC_{50} curve for the percentage of reduction of chemiluminescence of TMD-DBAS excitation vs logarithm of concentration of chlorogenic acid.

(C) TMD-DBAS chemiluminescence with respect to concentration of chlorogenic acid at different cycles of measurement (1 cycle = 63 s).

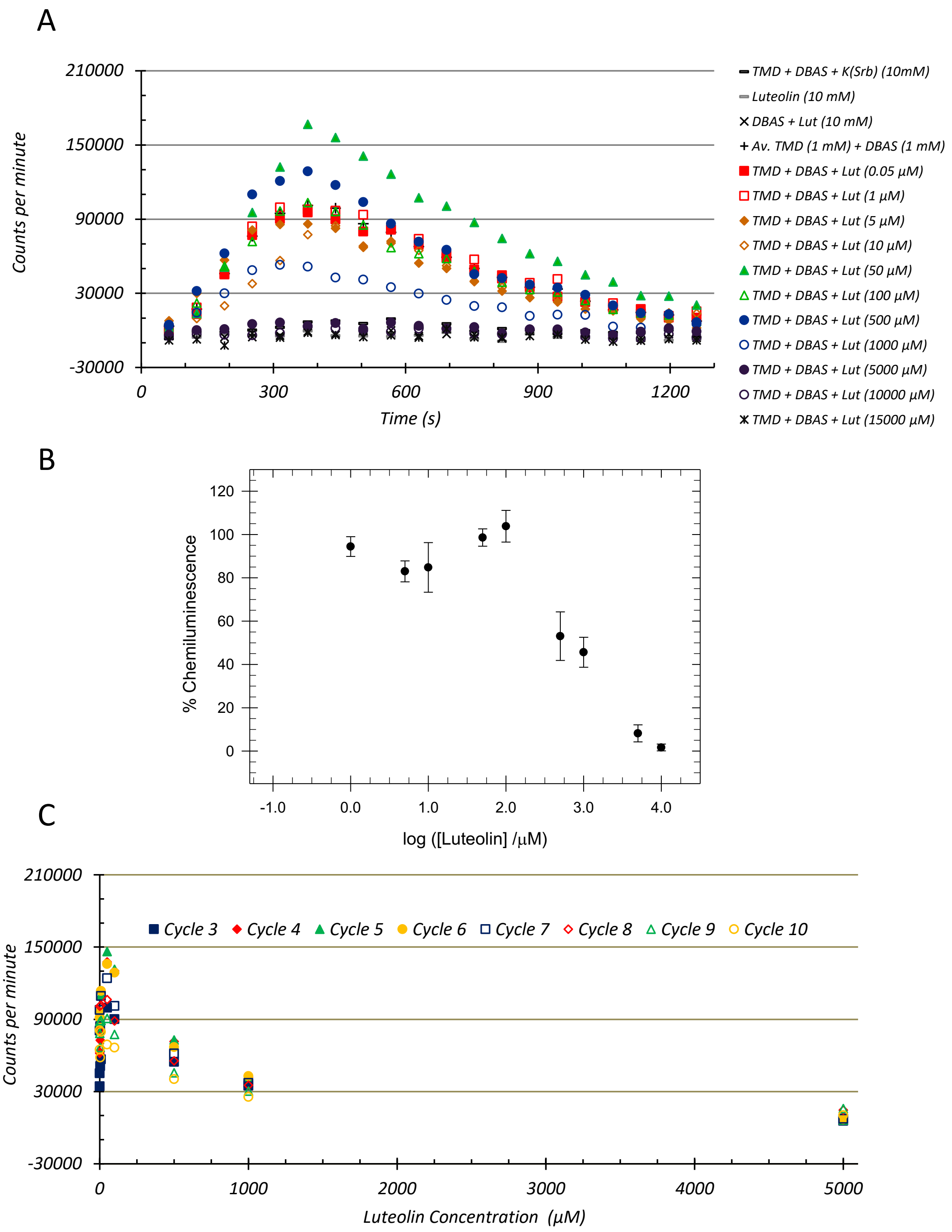

Figure S15. Chemiluminescence of TMD-DBAS excitation in the presence of **luteolin**.

(A) TMD-DBAS chemiluminescence with respect to time at different concentrations of luteolin.

(B) IC_{50} curve for the percentage of reduction of chemiluminescence of TMD-DBAS excitation vs logarithm of concentration of luteolin.

(C) TMD-DBAS chemiluminescence with respect to concentration of luteolin at different cycle of measurement (1 cycle = 63 s).

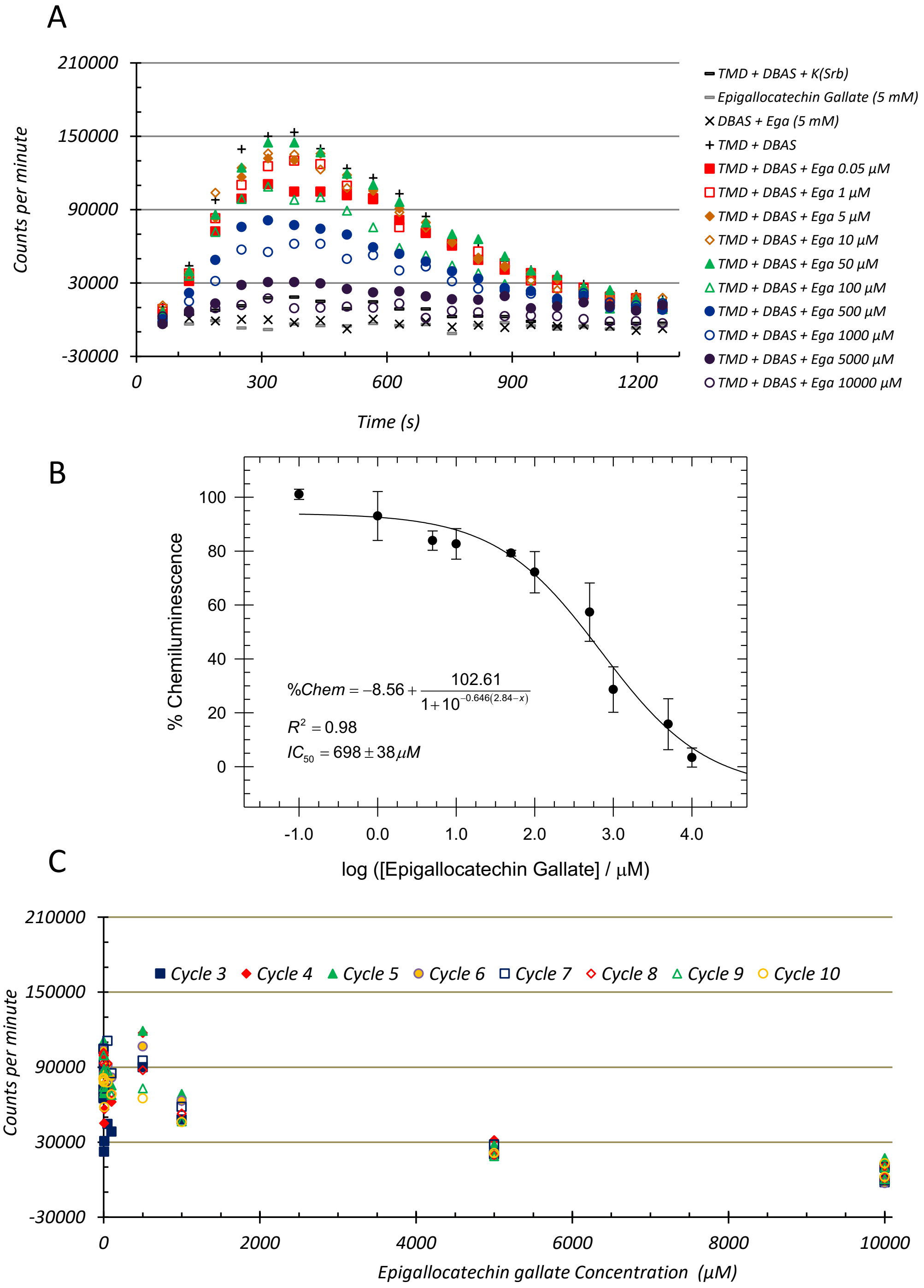

Figure S16. Chemiluminescence of TMD-DBAS excitation in the presence of **epigallocatechin gallate**.

(A) TMD-DBAS chemiluminescence with respect to time at different concentrations of epigallocatechin gallate.

(B) IC_{50} curve for the percentage of reduction of chemiluminescence of TMD-DBAS excitation vs logarithm of concentration of epigallocatechin gallate.

(C) TMD-DBAS chemiluminescence with respect to concentration of epigallocatechin gallate at different cycles of measurement (1 cycle = 63 s).

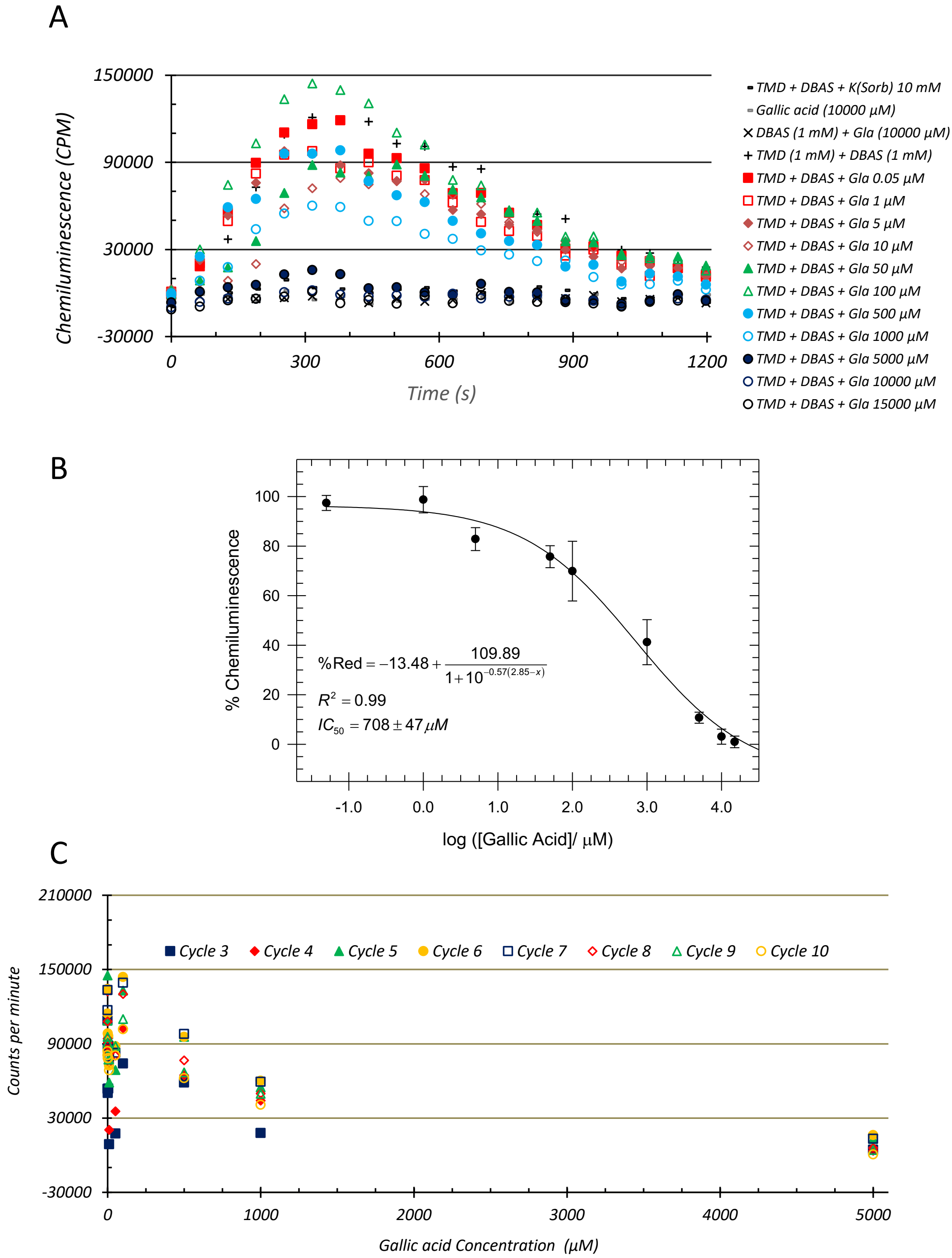

Figure S17. Chemiluminescence of TMD-DBAS excitation in the presence of **gallic acid**.

(A) TMD-DBAS chemiluminescence with respect to time at different concentrations of gallic acid.

(B) IC_{50} curve for the percentage of reduction of chemiluminescence of TMD-DBAS excitation vs logarithm of concentration of gallic acid.

(C) TMD-DBAS chemiluminescence with respect to concentration of gallic acid at different cycles of measurement (1 cycle = 63 s).

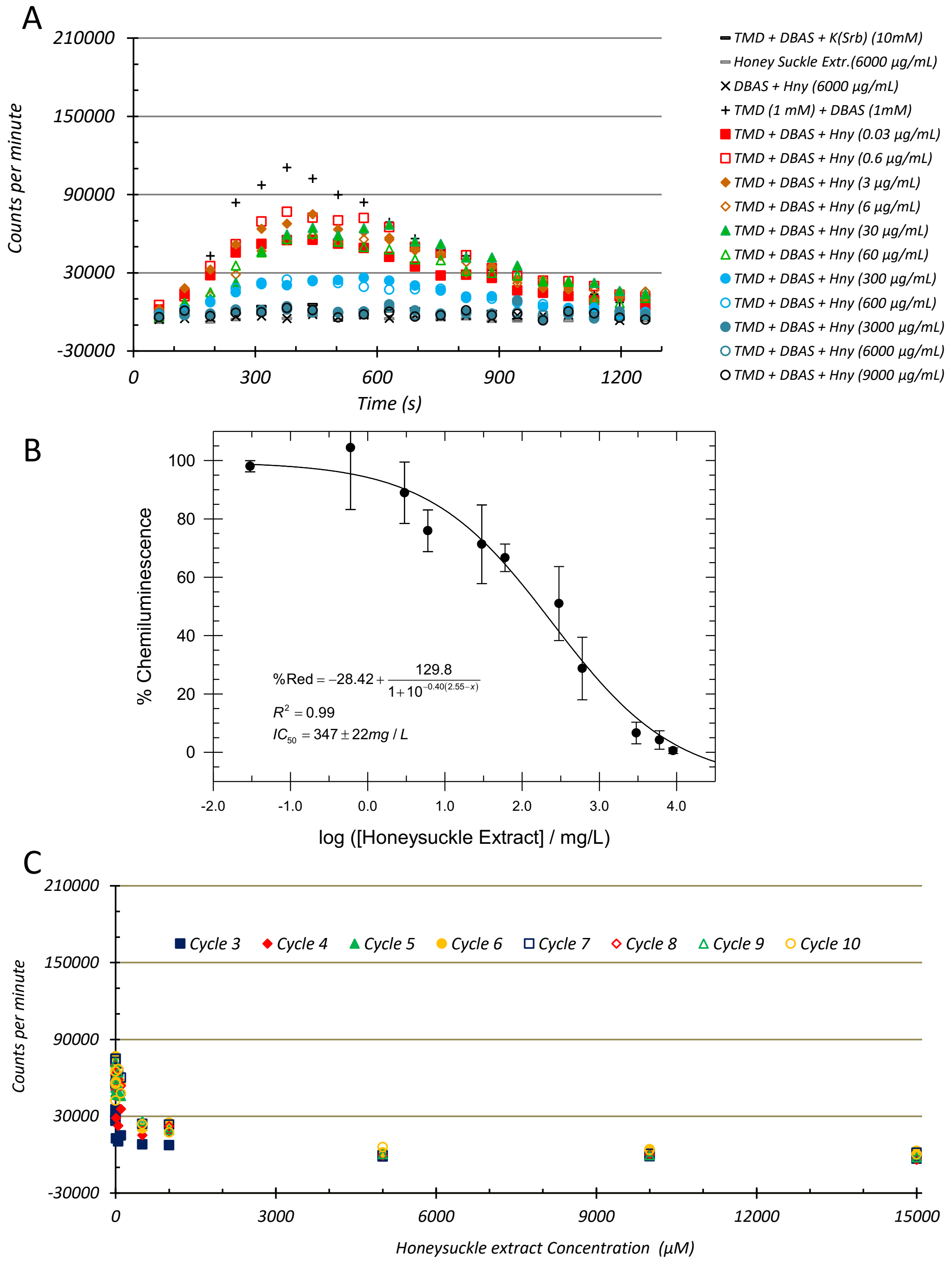

Figure S18. Chemiluminescence of TMD-DBAS triplet excitation in the presence of **honeysuckle extract**.
(A) TMD-DBAS chemiluminescence with respect to time at different concentrations of honeysuckle extract.
(B) IC_{50} curve for the percentage of reduction of chemiluminescence of TMD-DBAS excitation vs logarithm of concentration of honeysuckle extract.
(C) TMD-DBAS chemiluminescence with respect to concentration of honeysuckle extract at different cycles of measurement (1 cycle = 63 s).

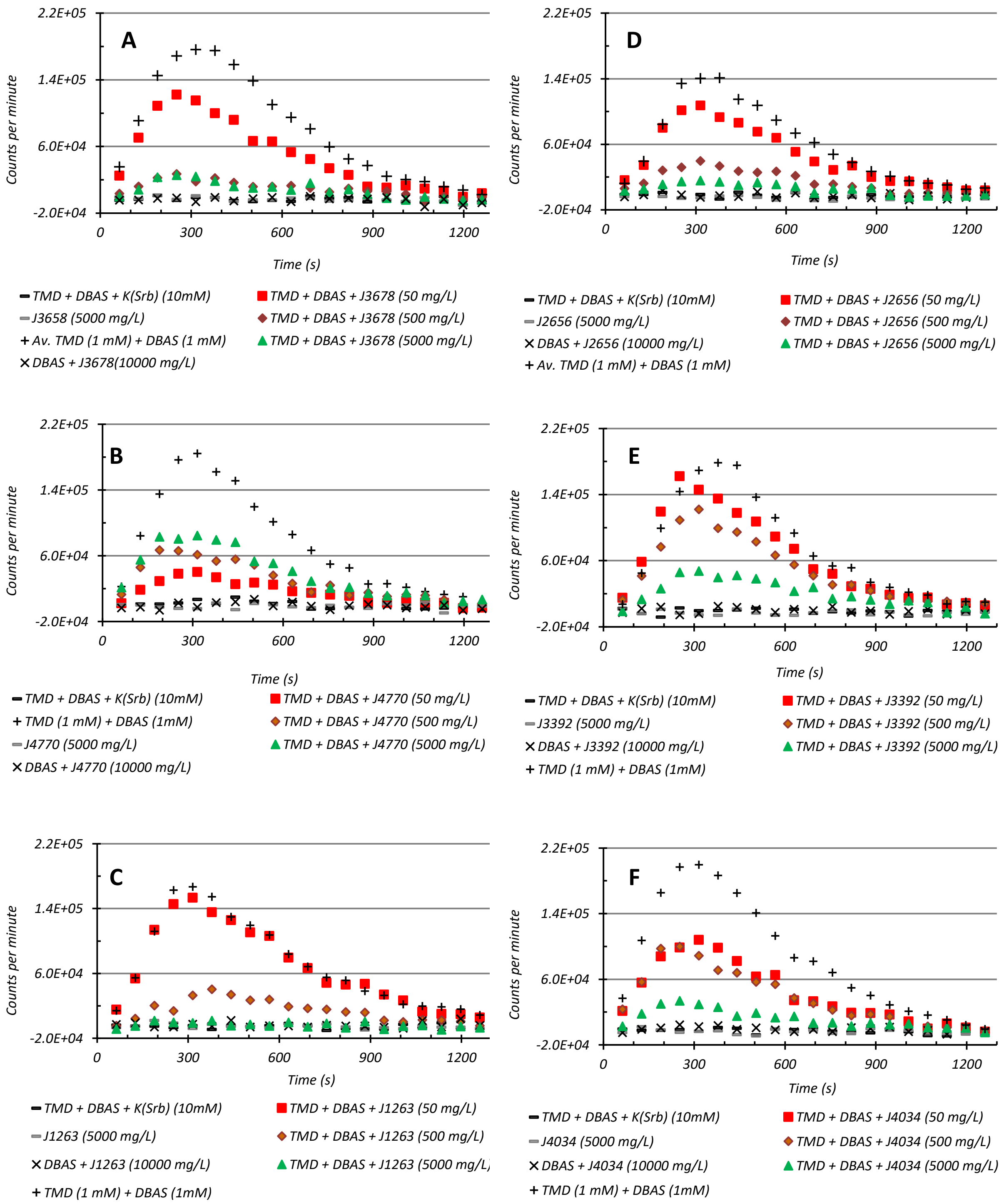

Figure S19. Chemiluminescence of TMD-DBAS triplet excitation in the presence of **algae extracts (A)** J3678, **(B)** J4770, **(C)** J1263, **(D)** J2656, **(E)** J3392, and **(F)** J4034.

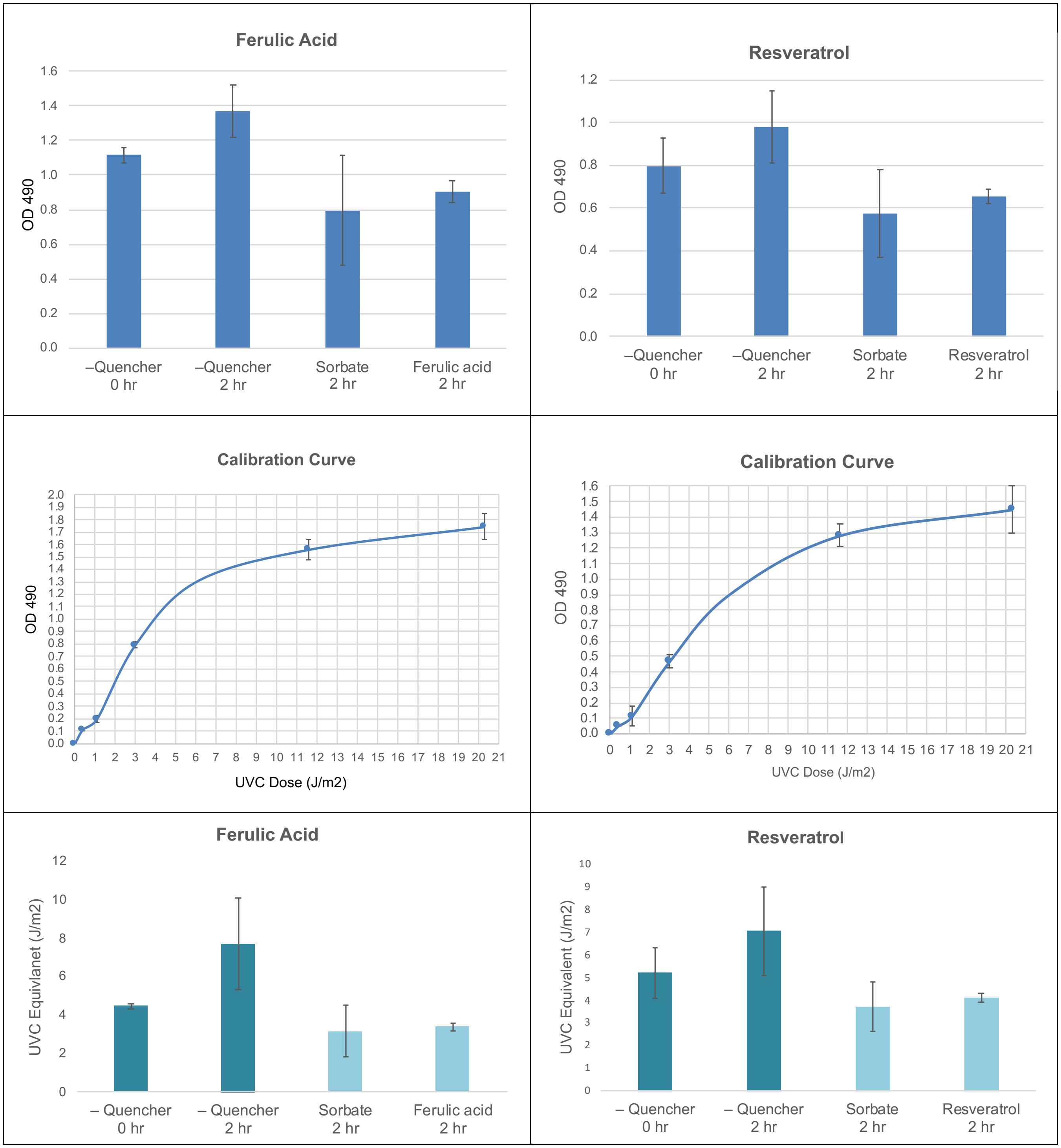
Ferulic Acid
OD 490
1.6
1.4
1.2
1.0
0.8
0.6
0.4
0.2
0.0
–Quencher 0 hr
–Quencher 2 hr
Sorbate 2 hr
Ferulic acid 2 hr
Resveratrol
OD 490
1.2
1.0
0.8
0.6
0.4
0.2
0.0
–Quencher 0 hr
–Quencher 2 hr
Sorbate 2 hr
Resveratrol 2 hr
Calibration Curve
OD 490
2.0 1.9 1.8 1.7 1.6 1.5 1.4 1.3 1.2 1.1 1.0 0.9 0.8 0.7 0.6 0.5 0.4 0.3 0.2 0.1 0.0
0 1 2 3 4 5 6 7 8 9 10 11 12 13 14 15 16 17 18 19 20 21
UVC Dose (J/m2)
Calibration Curve
OD 490
1.6 1.5 1.4 1.3 1.2 1.1 1.0 0.9 0.8 0.7 0.6 0.5 0.4 0.3 0.2 0.1 0.0
0 1 2 3 4 5 6 7 8 9 10 11 12 13 14 15 16 17 18 19 20 21
UVC Dose (J/m2)
Ferulic Acid
UVC Equivlanet (J/m2)
12
10
8
6
4
2
0
– Quencher 0 hr
– Quencher 2 hr
Sorbate 2 hr
Ferulic acid 2 hr
Resveratrol
UVC Equivalent (J/m2)
10
9
8
7
6
5
4
3
2
1
0
– Quencher 0 hr
– Quencher 2 hr
Sorbate 2 hr
Resveratrol 2 hr

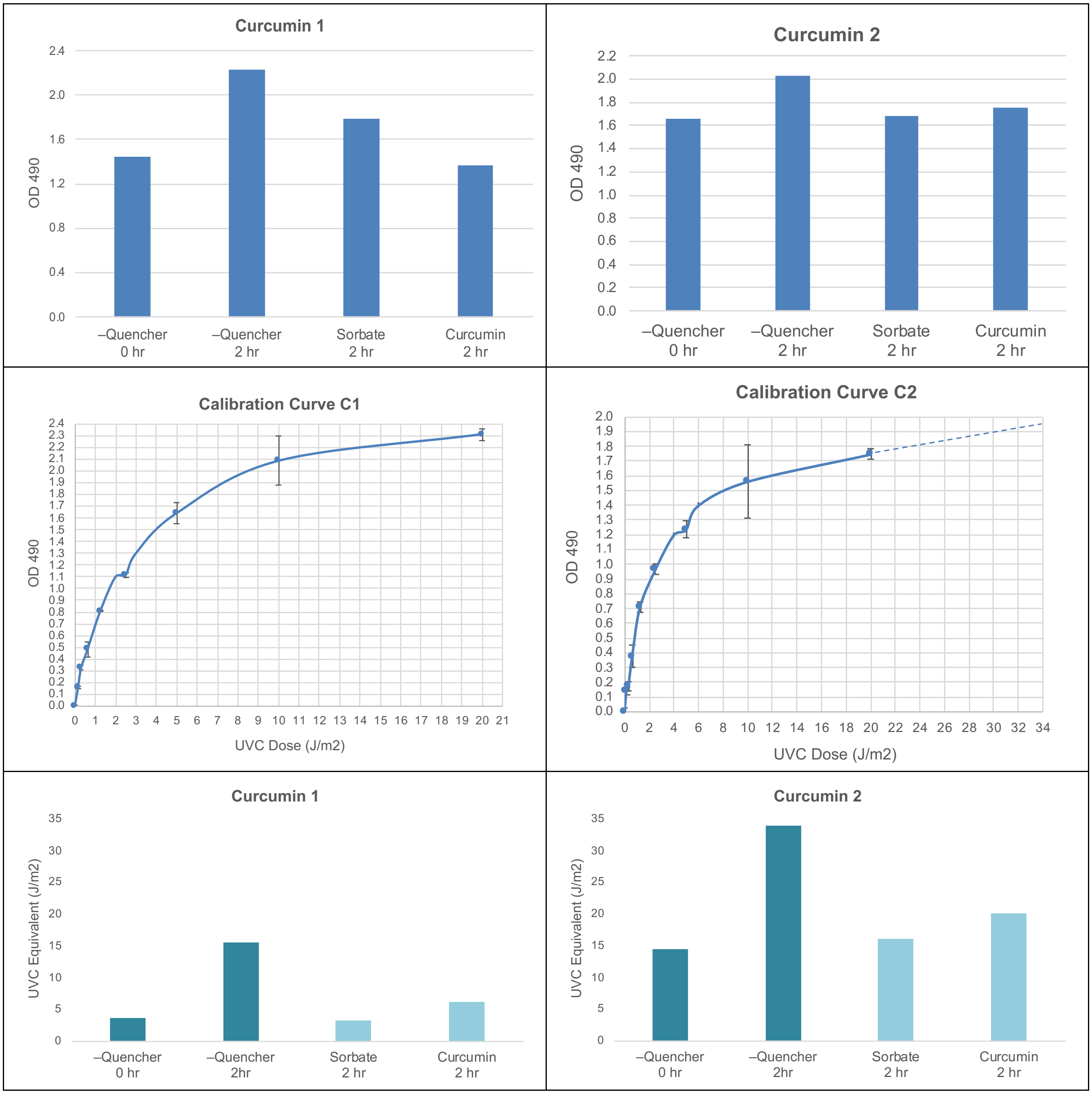

Figure S20. dCPD quenching activity before and after correcting for ELISA assay nonlinearity by using the UVC calibration curve.

C57BL/6 murine melanocytes were exposed in PBS to 75 kJ/m2 UVA, the PBS was replaced with medium, and 0 or 100 μM quencher added. Because the ELISA immunoassay for CPDs is non-linear, the ELISA signals for each experiment were calibrated in terms of equivalent UVC dose using a calibration curve constructed from UVC-irradiated DNA analyzed on the same ELISA plate on the same day.

(Top 3 rows, left) Ferulic acid and potassium sorbate positive control.

(Top 3 rows, right) Resveratrol and potassium sorbate.

(Lower 3 rows) Curcumin and potassium sorbate. Error bars reflect repeats performed on separate days.

Table S1. Triplet-state quenching survey.

	**DBAS Assay:**		**CPD Assay:**					
**Compound**	**TMD-DBAS Chemilum I** detects triplet quenching or dioxetane scission	**ONOO/melanin Chemilum** detects triplet quenching, dioxetane scission, or radical/ONOO scavenging	**ONOO/melatonin dCPD I ELISA-OD** detects triplet quenching, dioxetane scission, radical/ONOO scavenging, or CPD splitting	**ONOO/melatonin dCPD II ELISA-OD** (as for dCPD I)	**ONOO/melatonin dCPD III ELISA-Dynalight** (as for dCPD I)	**ONOO/drug-alone dCPD ELISA-OD** detects cmp enhancement of DBAS energy transfer or dioxetane on cmp	**UVA dCPD in melanocytes I** (as for dCPD I, plus cell uptake)	**UVA dCPD in melanocytes II w/ standard curve** (as for melano I)
	aqueous	*DMSO*	*aqueous*	*aqueous*	*aqueous*	*aqueous*	*aqueous*	*aqueous*
DBAS				++		XX	++	
curcumin	interpolation ~100 uM	+++	++	++	+	XX	no effect	+++
resveratrol	~300	XX		++		X		+++
sorbic acid	~300	++	+	+	++	X	no effect	
ethyl sorbate							++	
potassium sorbate								+++
ferulic acid		++	++	+	++	X	+	
polydatin	~200	little effect		+		no CPDs	no effect	
baicalin	~300	+++		++ (only if fresh)	++	no CPDs	no effect	
ellagic acid		+++	+		+	no CPDs	no effect	
Trolox	~600							
luteolin		++ (X at low conc)		XX	+++	XX		
epigallocatechin gallate		++			+	XXX		
retinol (vitamin A)	~300 (poor solubility)	X				no CPDs		
β-carotene	~300 (poor solubility)	+ (poor solubility)				no CPDs		
squalene	~300	little effect	no effect			no CPDs		
dipicolinic acid		+++				XX		
quinolinic acid				+		~no CPDs	+	
Honeysuckle extract (chlorogenic acids, rutin, quercetin, and cyanidin-3-glucoside)		little effect				no CPDs		
Ampelopsis extract (90% dihydromyricetin)		XXX				no CPDs		
Acacia catechu extract		XX				no CPDs		
**Key:**								
hit candidate		DMSO ≠ aqueous				quenches more dCPDs than it generates		
		X = *increases* chemilum		X = *increases* dCPD		X = *increases* dCPD		
		Enhances energy transfer						
		& light from triplet-state or						
		cmp forms dioxetane						

Table S2. TMD-DBAS quenching activity of algal extracts.

Natural Products ID	Country of Extraction	Genus	Species	Extract Type	Triplet Quenching Activity
J003678	Mauritius	*Acanthophora*	*muscoides*	Aqueous	+++
J004770	Florida	*Gracilaria*	*vermiculophylla*	Aqueous	++
J001263	Southern New Zealand	*Porphyra*	*sp.1*	*Organic*	++
J002656		*Acanthophora*	*muscoides*	Aqueous	++
J003392	Tanzania (Tanganyika, Zanzibar)	*Gracilaria*	*sp.*	Aqueous	++
J004034	Florida	*Gracilaria*	*foliifera*	Aqueous	++
J001348	Philippine islands	*Gracilaria*	*salicornia*	Aqueous	+
J001386	Philippine islands	*Gracilaria*	*arcuata*	Aqueous	+
J001654	Philippine islands	*Acanthophora*	*sp.*	Aqueous	+
J001970	Philippine islands	*Gracilaria*	*eucheumoides*	Aqueous	+
J002936	Celebes	*Gracilaria*	*sp.*	Aqueous	+
J003134	Palau islands	*Gracilaria*	*eucheumoides*	Aqueous	+
J003160	Palau islands	*Gracilaria*	*arcuata*	Aqueous	+
J003536	Tonga	*Gracilaria*	*edulis*	Aqueous	+
J003650	Mauritius	*Gracilaria*	*arcuata*	Aqueous	+
J003660	Mauritius	*Porphyra*	*tenera*	Aqueous	+
J003688	Mauritius	*Gracilaria*	*salicornia*	Aqueous	+
J003744	Vanuatu (New Hebrides)	*Gracilaria*	*salicornia*	Aqueous	+
J003938	Florida	*Gracilaria*	*tikvahiae*	Aqueous	+
J004050	Florida	*Gracilaria*	*damaecornis*	Aqueous	+
J004066	Malaysia	*Gracilaria*	*blodgettii*	Aqueous	+
J004070	Malaysia	*Gracilaria*	*gigas*	Aqueous	+
J004110	Palau islands	*Gracilaria*	*salicornia*	Aqueous	+
J004112	Palau islands	*Gracilaria*	*edulis*	Aqueous	+
J004274	Malaysia	*Acanthophora*	*spicifera*	Aqueous	+
J004356	Florida	*Gracilaria*	*blodgettii*	Aqueous	+
J004884	Florida	*Gracilaria*	*lemaneiformis*	Aqueous	+
C019162	Papua-New Guinea	*Acanthophora*	*spicifera*	Aqueous	–
J000784	Southeastern Australia	*Gracilaria*	*secundata cf*	Aqueous	–
J001358	Philippine Islands	*Gracilaria*	*crassa*	Aqueous	–
J002878	Mariana Islands (guam)	*Acanthophora*	*spicifera*	Aqueous	–
J003000	Palau Islands	*Gracilaria*	*cf. edulis*	Aqueous	–
J003078	Philippine Islands	*Gracilaria*	*edulis*	Aqueous	–
J000312		*Palmaria*	*palmata*	Aqueous	–
J000330		*Palmaria*	*mollis*	Aqueous	–
J001650	Philippine Islands	*Gracilaria*	*sp.*	Aqueous	–
J001676	Southwestern Australia	*Gracilaria*	*secundata*	Aqueous	–
J003504	Philippine Islands	*Acanthophora*	*muscoides*	Aqueous	–
J004372	Florida	*Gracilaria*	*blodgettii*	Aqueous	–
J004694	Puerto Rico	*Gracilaria*	*cuneata*	Aqueous	–
J004704	Puerto Rico	*Gracilaria*	*mammillaris*	Aqueous	–
J004708	Puerto Rico	*Acanthophora*	*spicifera*	Aqueous	–
J004716	Puerto Rico	*Gracilaria*	*cervicornis cf.*	Aqueous	–
J004728	Puerto Rico	*Gracilaria*	*verrucosa*	Aqueous	–
J004782	Florida	*Gracilaria*	*cylindrica*	Aqueous	–
