# Supplementary material for: Triplet-Energy Quenching Functions of Antioxidant Molecules
Source: Antioxidants (Basel). 2022 Feb 11;11(2):357. doi: 10.3390/antiox11020357 (PMC8868474; doi:10.3390/antiox11020357)
Supplement: Supplementary file 1 [file antioxidants-11-00357-s001.zip › antioxidants-1520830-supplementary methods.pdf]

SUPPLEMENTARY METHODS

Triplet-Energy Quenching Functions of Antioxidant Biomolecules

Carlos Angelé-Martínez, Leticia C. P. Goncalves, Sanjay Premi, Felipe A. Augusto, Meg A. Palmatier,

Saroj K. Amar, and Douglas E. Brash

Reagents

Caffeic acid (C0625), chlorogenic acid (C3878), Trolox (238813), potassium sorbate (359769), glutathione (G4251), hydrogen peroxide (8.22287), diethyl ether (without peroxide inhibitor, 309966), 2,3-dimethyl-2-butene (220159), 1,3-dibromo-5,5-dimethylhydantoin (157902), silver acetate (216674), Tween-20 (P7949), Trizma (T2663), diethylenetriamine pentaacetic acid (DTPA, 32319), and calf thymus DNA (D1501) were purchased from Sigma. Magnesium sulfate (M65-500), sodium bicarbonate (S233-500), ammonium sulfate (AAA116820I), pentane (P399-4), and dichloromethane (D143SK-4) were purchased from Fisher Scientific. 9,10-dibromoanthracene-2-sulfonic acid sodium salt (SC210717) was purchased from SantaCruz Biotechnology. Sodium bicarbonate (3506-01) and sodium hydroxide (3722-01) were purchased from VWR. Solutions of NaCl (5M, AB13198), $MgCl_2$ (1 M, AB09006), and TE (1X) were purchased from AmericanBio. Resveratrol, ferulic acid, polydatin, ellagic acid, curcumin, baicalein, baicalin, catechin, myricetin, luteolin, epigallocatechin gallate, and gallic acid, each of >95% purity, and a polyphenolic mixture (honeysuckle extract) were provided by L'Oreal, Inc. (Clark, NJ). All chemicals were used without further purification. Solutions were freshly prepared prior to use in deionized water (18 mΩ, MilliQ, IQ 7000). The algae extracts were a kind gift of the Natural Products Branch, National Cancer Institute.

Single-stranded oligo dT_{100} and oligo dT_{150}, 100- and 150-mers of thymine, were synthesized by the Yale Keck Biotechnology Resource Laboratory. For the 150-mer, synthesis was performed on an ABI 394 oligo synthesizer with a Glenn Research twist DT column, using 4,5-dicyanoimidazole as synthesis activator, dichloroacetic acid as deblocker, 4-dimethylaminopyridine as capping reagent, and iodine (0.02 M) in 80%:10%:10% tetrahydrofuran:pyridine:acetic anhydride as oxidizer. Deprotection of the phosphate groups was performed by treating the 150-mer with 10% diethanolamine in acetonitrile to prevent the cyanoethyl-base byproduct formed upon treatment with a stronger base (e.g. ammonium hydroxide) [1]. After the deprotection step, the 150-mer was cleaved from the solid phase using ammonium solution (30%) in methyl amine and then desalted using acetonitrile and eluted in ammonium hydroxide (20% in water).

Synthesis of TMD

The synthesis of tetramethyldioxetane (TMD) was performed by the procedure of Kopecky, et al. [2,3], in two major steps as described below. Before carrying out the synthesis, we advise researchers to read the original papers to become familiar with the potential explosion hazards.

Synthesis of bromohydroperoxide.

Hydrogen peroxide (50% v/v, 24 mL) was extracted in diethyl ether (2×20 mL). The organic layer was dried with $MgSO_4$ overnight at 4°C and the resulting concentration of H_2O_2 was measured by UV absorption at 240 nm. Ethereal H_2O_2 (250 mmol) and 2,3-dimethyl-2-butene (50 mmol, 5.9 mL) were added to a 3-neck round bottom flask and cooled down at -40 °C under nitrogen atmosphere and mechanical stirring. Over a period of two hours,

1,3-dibromo-5,5-dimethylhydantoin (20 mmol, 5.7 g) was added, after which the -40°C bath was replaced by an ice-water bath. The progress of the reaction was monitored by thin layer chromatography on a SiO_2 matrix (R_f = 0.58) with 254 nm UV detection. After one hour in the ice-water bath, the reaction mixture was washed with cold $NaHCO_3$ (10% w/v in water, 2 × 20 mL), cold $(NH_4)_2SO_4$ (10%, 2×20 mL), and cold water (20 mL). The removal of H_2O_2 was checked by TLC (R_f=0) and if incomplete the washings must be repeated. The light-yellow organic phase was dried with anhydrous $MgSO_4$ overnight at 4°C. The solution was filtered through Whatman filter paper (grade 201, WHA 5201090, Sigma) at 0°C, and later the solvent was evaporated at reduced pressure on an ice-water bath. The white solid was recrystallized from pentane at 4 °C. The pentane was removed and the recrystallized solid was air-dried at room temperature over an ice-water bath.

Cyclization of TMD.

A suspension of silver acetate (2.5 g, 0.015 mol) in dichloromethane (30 mL) was placed in a 3-neck rounded bottom flask with a drying tube and over an ice - water bath. The previously synthesized bromohydroperoxide (1 g, 0.005 mol) was dissolved in dichloromethane (20 mL) and added dropwise under N_2 atmosphere and magnetic stirring (>500 rpm) over about 1 h. The ice-water bath was removed and the reaction continued at room temperature. After 20 min., the reaction mixture was filtered through sintered glass. The pale-yellow filtrate was washed with Na_2CO_3 (10%, 1×20 mL) and dried with $MgSO_4$ for 5 min.

The $MgSO_4$ was gravity filtered and the organic solvent was evaporated at reduced pressure over an ice bath. Pentane was added to the yellow oil and the mixture incubated at 4°C for 24 h to recrystallize the TMD. The pentane was removed, and the crystals (needles) were dried and weighed. A solution of TMD (200 mM) in dimethoxyethane was prepared for future use.

Quenching of Triplet-Reporter Luminescence by Polyphenols and Related Compounds

Resveratrol and curcumin were dissolved in ethanol-water (50%). Ellagic acid, myricetin, baicailin, and luteolin were dissolved by adding NaOH (1 M, <5% total volume) and ethanol 25%. DBAS was dissolved in DMSO. All other compounds and the honeysuckle and powdered algae extracts were completely dissolved in deionized water. TMD-DBAS-polyphenol chemiluminescence assays were performed in 300 µL total reaction volume. Samples were prepared by mixing phosphate buffer (pH 7, 25 mM), DTPA (0.2 mM), the natural compounds solution at various concentrations (0.05 – 15000 µM), TMD (1 mM), and DBAS (1 mM). DTPA was used as metal chelator because metal ions catalyze the decomposition of TMD [4]. Samples were aliquoted (100 µL) in duplicate into white 96-well plates Greiner Lumitrac 200 (Fisher Scientific, 655075) and the chemiluminescence was measured in a FluoDia T70 Microplate Photon Counting Fluorometer (Photal Otsuka Electronics), using 300 ms as integration time, and 73 °C as nominal temperature.

In early surveys (**Table S1**), reactions contained 50 µg/mL synthetic tyrosine-derived melanin (Sigma M8631), 200 mM $ONOO^-$ (stock in 0.2M NaOH, Millipore 516620), 50 mM DBAS, and 1 mM pure quencher (stock in DMSO) or 100 µg/mL of extract; all reactions were performed in DMSO. After mixing, the sample was immediately inserted into a Tri-Carb Liquid Scintillation Analyzer (Perkin-Elmer) set to Single Photon Counting mode and counted every 25 s for 10 min using Quanta Smart analysis software (Perkin-Elmer).

Quenching of Dark Cyclobutane Pyrimidine Dimer Formation

In a microcentrifuge tube, 1 µg of dT_{150} or calf thymus DNA (^{CT}DNA) in phosphate buffer (pH 7, 50 mM) and DTPA (0.5 mM) was treated with TMD (5 mM) and freshly prepared solutions of epicatechin, potassium sorbate, or luteolin (10 µM) in a total volume of 200 µL. The solution with all reagents was vortexed briefly and incubated at 70 °C for 10 min. The samples were purified by column elution. To assess triplet-quenching in cells, C57BL/6 murine melanocytes were exposed to 75 kJ/m^2 UVA as previously reported [5], 100 µM quencher added, and cells harvested at 2 h.

In early surveys (**Table S1**), 2 µg of dT_{100} was incubated with 4 additions of reagents over 1.5 h, summing to 0.25 mM melatonin [6], 1.5 mM $ONOO^-$, 2 mM quencher, in 50 mM phosphate buffer pH 7.4 (n = 2). In those experiments, cells were incubated in 10 mM test compound after exposure to 75 kJ/m^2 UVA and harvested at 1 and 2 h for dCPD analysis.

Column Purification of dT_{150}

Purification of dT_{150} was performed using ZYMO Oligo Clean and Concentrator (Research Products International, RPI, ZD4060) following the manufacturer's protocol. Briefly, 400 µL of oligo binding buffer was added to the dT_{150} solution (200 µL, 100 µL of buffer per 50 µL of sample) and the mixture vortexed briefly. Ethanol (800 µL) was added and vortexed briefly. The ethanolic mixture was transferred to a column and centrifuged (12,200 g, 40 s). The ethanolic solution was transferred to the column in two steps as the column capacity is ~900 µL. The flowthrough was discarded. DNA wash buffer (750 µL) was then added to the column and the column was centrifuged (12,200 g, 40 s). The flowthrough was discarded and the column was transferred into a 1.5 mL microcentrifuge tube. The dT_{150} was eluted by adding 55 µL of TE buffer (1X, AmericanBio) to the center of the column and centrifuging it (14,000 rpm, 40 s). Samples were stored at 4°C. Absorbance of the purified samples was measured in duplicate by Nanodrop instrument (ND-1000, Thermo-Fisher) (1.5 – 2.0 µL each) in the single stranded DNA mode using TE (1X) as blank.

Column Purification of ^{CT}DNA

^{CT}DNA treated with TMD and the triplet quenchers was purified using the ZYMO Genomic DNA Clean and Concentrator column (Research Products International, ZD4064) following the manufacturer's protocol. To 200 µL of ^{CT}DNA was added 3 volumes of ChIP DNA binding buffer (600 µL). The buffered solution was transferred to a Zymo-Spin^{TM} IIC-XL Column and centrifuged (12,200 g, 40 s). The flowthrough was discarded, the column was washed with DNA wash buffer by centrifugation (12,200 g, 40 s), and the flowthrough discarded. Washing was done twice. The column was air dried for 1 min and then transferred to a 1.5 mL microcentrifuge tube. DNA Elution Buffer (50 µL) was added to the center of the column matrix and incubated for 5 min at room temperature. The ^{CT}DNA was eluted by centrifugation (12,200 g, 40 s). Sample concentration was measured by Nanodrop in duplicate (1.5 µL each) and stored at 4 °C for later CPD detection.

Detection of Cyclobutane Pyrimidine Dimers by Enzyme Linked Immunosorbent Assay (ELISA)

Antibodies and buffers.

TBS (1X) buffer solution was prepared by dissolving Tris buffer (1X, 54.4 mL), and NaCl solution (5M, 32.6 mL) in deionized H_2O. The washing buffer (TBST) was prepared as TBS and adding Tween-20 (544 µL). Normal Goat Serum (GIBCO, 16210-064, 1.2 mL) was diluted in TBS (1X, 60 mL) to obtain a 2% solution. Carbonate buffer was prepared by dissolving $NaHCO_3$ (0.84 g) in deionized H_2O (90 mL), a solution of $MgCl_2$ (1M, 1 mL) was added, and the pH was adjusted to 9.5 by adding NaOH solution (0.1 mM). The volume was increased to 100 mL by adding deioinized H_2O. The primary antibody (CosmoBio Co., TMD-2, NMDND001) was first dissolved in deioinized H_2O (100 µl), and then 12 µL were diluted 1000 fold in 2% NGS. The secondary antibody linked to alkaline phosphatase, GTXMUIgG F(ab')-2 AP x ADS (ThermoFisher Scientific, A24527) was diluted 1000-fold in 2% NGS.

Denaturation and plating of dT_{150} and ^{CT}DNA.

From samples treated with TMD and column purified, or UVC irradiated, a 3 ng/µL solution (160 µL) was prepared. The samples were denatured at 95°C for 15 min and then transferred to an ice-water bath for 15 min. The samples were aliquoted in triplicate (50 µL) into wells of a 96-well, non-binding Greiner plate (E19033A5)

that had been pre-washed 6 times with TBS (1X), Tween (0.05%, see above) and then 3 times with deionized H_2O. The plate was incubated at 37°C overnight.

Antibody incubation and visualization.

The plate was washed 5 times with Tris buffered saline-Tween solution (TBST 1X), discarding the buffer and wiping the plate onto an absorbing pad after each wash. The plate and oligo were blocked with 300 µL of 2% NGS in Tris buffered 1X TBS by incubating the solution at 37°C for 30 min. The NGS solution was discarded and the plate wiped.

Primary antibody, TDM-2, in TBS (1X) was added (100 µL) to each plated sample well and incubated at 37°C for 30 min. The plate was washed 5 times with TBST (1X), discarding the buffer and wiping the plate onto an absorbing pad after each wash. Secondary antibody in TBS (1X) was added (100 µL) to the plated samples and incubated at 37°C for 30 min. The plate was washed 5 times with TBST (1X) and once more with carbonate buffer, with the buffer discarded, and the plate wiped onto an absorbing pad after each wash.

One hundred microliters of Dynalight solution (Thermo Fisher Scientific, Dynalight Substrate with RapidGlow Enhancer, 4475406) were added to the plated samples and incubated at room temperature for 30 min. Fluorescence of the samples was measured in a FluoDia T70 Microplate Photon Counting Fluorometer (Photal Otsuka Electronics), using 300 ms as integration time at room temperature.

Calibration Curve for Quantifying CPD Generated by Chemiexcitation

The CPD ELISA gives a nonlinear dose response, so ELISA values were converted to physical values related to CPD density. We used UVC to create DNA samples with a known and well-characterized series of CPD densities and having a minimum of other DNA lesions. A solution of dT_{150} (10 ng/µL, 2.5 mL) was prepared from the solid stock. The solution was split into 10 µL drops on Parafilm on a cold block. The drops were irradiated with a UVC lamp calibrated with a UVX Radiometer (Analytik Jena, UVP 97001502) and a UVC probe (Analytik Jena, UVX-25) for a time providing a dose of 100 J/m^2. After irradiation, the sample was column purified as in section 2.5. Similarly, a non-irradiated solution of dT_{150} (10 ng/µL, 8 mL) was column purified. The concentration of purified solutions was measured in duplicate (1.5 µL) by Nanodrop as above. Both solutions were diluted to 3 ng/µL and the irradiated solution was mixed with the non-irradiated solution at different ratios to provide dT_{150} equivalent to irradiation at different irradiation doses. These calibration standards were used within each experiment to generate ELISA calibration curves. The curves were used to convert ELISA values to their UVC Equivalent dose (in J/m^2). A UVC dose of 5 J/m^2, the approximate value seen in the 0 hr controls, generates ~1 CPD per 10 kbp [7].

Quantification of Quenching

Biochemical studies.

To calculate IC_{50} values for inhibiting TMD-DBAS luminescence, the average luminescence signal of the non-chemiluminescent wells containing no TMD or DBAS was subtracted from the chemiluminescence signal of each experimental well containing TMD, DBAS, and the putative quencher, with the result denoted S_{TDQ}. Luminescence from TMD or DBAS alone was not significant. Percent Chemiluminescence was defined as the ratio of luminescence in the presence of quencher to that in the absence of quencher, 100 * (S_{TDQ}/S_{TD}). Luminescence inhibition is then 100 - S_{TDQ}/S_{TD}. The IC_{50} value was calculated by plotting %Chemiluminescence versus the logarithm of the concentration of the triplet quencher concentration (in µM) and fitting to a variable slope sigmoidal dose-response curve using SigmaPlot version 11.0 (Systat Software, Inc., San Jose, CA). Average IC_{50} values and IC_{50} experimental errors reported in **Table 1** are calculated from at least three independent experiments. The percent CPD inhibition was calculated as 100 - % CPD increase. Because the DNA-alone ELISA signal was not negligible, % CPD increase was calculated as 100 * (S_{TDQ} - S_D)/(S_{TD} - S_D) and D now stands for DNA-

alone rather than DBAS. The standard deviation of the quenching was calculated from two independent experiments.

Cell-based studies.

The scale of CPD production at 0 h and of dCPD production at 2 h both varied between experiments. Cell-based UVC Equivalent dose were therefore normalized to the value at 0 h. Pre-normalized data are presented in **Figure S20**.

References

1. atdBio. Solid-Phase Oligonucleotide Synthesis. https://atdbio.com/nucleic-acids-book/Solid-phase-oligonucleotide-synthesis (accessed on 11 December 2021).
2. Kopecky, K.R.; Filby, J.E.; Mumford, C.; Lockwood, P.A.; Ding, J.Y. Preparation and thermolysis of some 1,2-dioxetanes. *Can. J. Chem.* **1975**, *53*, 1103–1122.
3. Baader, W.J.; Bastos, E.L. Four-membered cyclic peroxides (1,2-dioxetanes and 1,2-dioxetanones). *Sci. Synth.* **2009**, *38*, 323–344.
4. Hardeland, R.; Tan, D.X.; Reiter, R.J. Kynuramines, metabolites of melatonin and other indoles: The resurrection of an almost forgotten class of biogenic amines. *J. Pineal Res.* **2009**, *47*, 109–126.
5. Premi, S.; Wallisch, S.; Mano, C.M.; Weiner, A.B.; Bacchiocchi, A.; Wakamatsu, K.; Bechara, E.J.; Halaban, R.; Douki, T.; Brash, D.E. Photochemistry. Chemiexcitation of melanin derivatives induces DNA photoproducts long after uv exposure. *Science* **2015**, *347*, 842–847.
6. Hardeland, R.; Tan, D.X.; Reiter, R.J. Kynuramines, metabolites of melatonin and other indoles: The resurrection of an almost forgotten class of biogenic amines. *J. Pineal Res.* **2009**, *47*, 109–126.
7. Premi, S.; Han, L.; Mehta, S.; Knight, J.; Zhao, D.; Palmatier, M.A.; Kornacker, K.; Brash, D.E. Genomic sites hypersensitive to ultraviolet radiation. *Proc. Natl. Acad. Sci. USA* **2019**, *116*, 24196–24205.
